# Machine Learning, Large Language Models, and Multimodal AI for Diagnosing Pediatric Rare Diseases: Scoping Review

**DOI:** 10.2196/96169

**Published:** 2026-08-28

**Authors:** Jungang Zhao, Jiawei Luo, Qiu Li, Yaolong Chen

**Affiliations:** 1 Chevidence Lab Child & Adolescent Health, Department of Pediatric Research Institute, Children's Hospital of Chongqing Medical University, National Clinical Research Center for Children and Adolescents' Health and Diseases, Ministry of Education Key Laboratory of Child Development and Disorders, 136 Zhongshan Second StreetChongqing, 400014, China, +86 23 68370084; 2 Chongqing Municipal Health Commission Key Laboratory of Children's Vital Organ Development and Diseases, Chongqing, China; 3 Department of Nephrology, Children's Hospital of Chongqing Medical University, Chongqing, China; 4 Research Unit of Evidence-Based Evaluation and Guidelines, Chinese Academy of Medical Sciences (2021RU017), School of Basic Medical Sciences, Lanzhou University, Lanzhou, China; 5 WHO Collaborating Center for Guideline Implementation and Knowledge Translation, Lanzhou University, Lanzhou, China

**Keywords:** rare diseases, pediatrics, AI, machine learning, deep learning, large language models, multimodal AI, diagnostic decision support, scoping review

## Abstract

**Background:**

Pediatric rare diseases often cause a prolonged diagnostic odyssey. AI, including machine learning, deep learning, large language models (LLMs), and multimodal systems, may support diagnosis, but these applications in children have not been systematically mapped.

**Objective:**

The aim of the study is to map diagnostic applications, data modalities, validation strategies, and evidence maturity of AI methods for pediatric rare diseases.

**Methods:**

We conducted a scoping review following Joanna Briggs Institute methodology and reported it according to PRISMA-ScR (Preferred Reporting Items for Systematic Reviews and Meta-Analyses extension for Scoping Reviews). On June 26, 2026, we searched PubMed, Scopus, Web of Science Core Collection, Embase, China National Knowledge Infrastructure (CNKI), Wanfang Data, and the Cochrane Library for records published from January 1, 2015, through June 1, 2026. We additionally searched medRxiv and arXiv and hand-searched the reference lists of included studies and relevant reviews. Eligibility was defined using the population-concept-context framework: pediatric rare diseases, diagnostic AI, and any clinical or research setting. JZ and JL independently screened titles and abstracts and assessed potentially eligible full-text reports. JZ charted the data, and JL verified every field. Findings were synthesized descriptively according to disease focus, AI technology, input modality, diagnostic task, validation strategy, and evidence maturity.

**Results:**

Database searches identified 2557 records; 2063 remained after deduplication. Of 106 full-text reports assessed, 77 database studies and 4 studies from hand searching and preprint servers were included, yielding 81 studies. Studies were published from 2016 through 2026, with 55 of 81 (67.9%) published from 2024 through 2026. Using a mutually exclusive primary technology classification, classical machine learning accounted for 38 (46.9%) studies, facial AI for 18 (22.2%), deep learning for 15 (18.5%), LLMs for 6 (7.4%), and multimodal AI for 4 (4.9%). Electronic health records, claims, clinical text, or structured clinical vignettes were used in 25 (30.9%) studies, facial images in 17 (21%), and other medical imaging in 13 (16%). Evidence remained mainly retrospective and internally validated: 62 (76.5%) studies included a retrospective component and 76 (93.8%) reported internal validation, whereas 21 (25.9%) included external validation and 12 (14.8%) included a prospective component.

**Conclusions:**

Research on AI-assisted diagnosis of pediatric rare diseases has expanded rapidly, but evidence maturity has not kept pace. Most studies established technical feasibility rather than generalizable clinical benefit, and performance should be interpreted by task, inputs, reference standard, and validation design rather than used to rank technologies. Evidence for LLMs and multimodal AI remains limited. Future research should prioritize multicenter validation, reproducible task-specific benchmarks, prospective evaluation, and assessment of incremental clinical value.

## Introduction

Pediatric rare diseases pose a substantial challenge to health systems worldwide [1]. Although each condition affects relatively few individuals, rare diseases collectively represent a considerable burden, and most have a genetic origin [2]. Affected children frequently experience a prolonged diagnostic odyssey before receiving a definitive diagnosis. Diagnostic delay may result in repeated or unnecessary investigations, missed opportunities for timely management, delayed genetic counseling, and substantial psychological and financial burdens for families. Diagnosis is particularly difficult because individual conditions are uncommon, phenotypes frequently overlap, clinical manifestations may change with age, and access to specialized expertise is uneven across health systems. These challenges create a need for decision-support approaches capable of integrating heterogeneous clinical information and helping clinicians identify or prioritize rare-disease diagnoses more efficiently.

AI offers several potential approaches to this problem. Classical machine learning (ML) and deep learning (DL) have been applied to electronic health records, laboratory measurements, medical images, facial photographs, and genomic data for tasks including patient identification, diagnostic classification, variant prioritization, and facial phenotyping, as exemplified by Face2Gene (FDNA Inc) [3]. More recently, large language models (LLMs), including ChatGPT (OpenAI) and GPT-4, have been investigated for phenotype extraction, clinical information synthesis, differential-diagnosis generation, and reverse phenotyping from free-text records [4]. Multimodal AI extends these approaches by jointly processing at least 2 distinct data modalities, such as clinical narratives, imaging, laboratory findings, physiologic signals, facial images, or genomic data, within an integrated diagnostic model or pipeline.

However, the application of AI to pediatric rare diseases involves challenges that may not be adequately represented in studies of common diseases or adult populations. Pediatric phenotypes may be incomplete, age-dependent, or documented longitudinally across multiple encounters. Rare-disease datasets are often small, imbalanced, and concentrated in specialist centers, while confirmatory diagnoses may depend on genomic, biochemical, imaging, or pathological evidence that is not available at the time of initial suspicion. Facial, genomic, and language-based systems may also be sensitive to ancestry, population representation, documentation practices, and local diagnostic pathways. Consequently, favorable performance in a curated or internally validated dataset may not translate directly into generalizable clinical utility.

Previous reviews have addressed only parts of this rapidly evolving field. A scoping review of DL for rare diseases did not focus specifically on children and did not include LLMs [5]. Another scoping review examined ML applications in rare diseases but did not address LLMs or multimodal AI [6]. A review of LLMs for disease diagnosis was not restricted to pediatric populations or rare diseases [4]. A narrative review discussed AI in pediatric rare diseases, including LLMs, but did not follow a formal scoping or systematic review methodology [7]. To our knowledge, no previous scoping review has systematically mapped classical ML, DL, facial AI, LLMs, and multimodal AI across the range of diagnosis-related tasks in pediatric rare diseases.

A scoping review was appropriate because the available literature was expected to be heterogeneous in disease focus, AI technology, input modality, diagnostic task, study design, reference standard, validation strategy, and outcome measure [8-10]. This approach permits systematic charting of the extent and characteristics of the evidence while avoiding inappropriate pooling or indirect comparison of performance estimates derived from fundamentally different clinical tasks and datasets. It also enables evidence density to be distinguished from evidence maturity, including whether studies progressed from retrospective development and internal validation to independent external validation, prospective evaluation, or assessment in clinical workflows.

Accordingly, this scoping review aimed to map AI applications for diagnosis-related tasks in children with suspected or confirmed rare diseases. We examined the diseases and populations represented, AI technologies and input modalities used, diagnostic tasks addressed, reference standards and outcome measures applied, geographic settings, and validation strategies. We further assessed how reported performance aligned with the intended diagnostic task and whether the available evidence primarily supported technical feasibility, transportability across settings, or clinical utility.

## Methods

### Study Design, Review Question, and Reporting Framework

We conducted a scoping review to map the use of AI for diagnosis-related tasks in pediatric rare diseases. The review question was: What AI approaches have been developed or evaluated for pediatric rare-disease diagnosis, and what diseases, input modalities, diagnostic tasks, validation designs, performance measures, and evidence gaps are represented in the literature?

The review followed the Joanna Briggs Institute methodology for scoping reviews [8] and the framework described by Arksey and O’Malley [10]. Eligibility was structured using the population-concept-context framework, and reporting followed the PRISMA-ScR (Preferred Reporting Items for Systematic Reviews and Meta-Analyses Extension for Scoping Reviews) [9]. The completed PRISMA-ScR checklist is provided in Checklist 1.

### Protocol, Registration, and Amendments

The review was submitted to PROSPERO on February 25, 2026, and publicly registered on March 19, 2026 (CRD420261326146; version 1.0). Registration occurred after the initial database searches conducted on February 1, 2026, but before completion of study screening, data charting, and evidence synthesis. During paper revision, the protocol was amended to reflect the expanded final search strategy and the methods actually undertaken, including the addition of Embase, China National Knowledge Infrastructure (CNKI), Wanfang Data, the Cochrane Library, medRxiv, arXiv, and reference-list hand searching. The amended PROSPERO record was publicly updated as version 1.1 on July 27, 2026. Multimedia Appendix 1 documents the final search strategy and protocol amendments.

The main methodological amendments from version 1.0 to the final review were expansion of search sources, extension of the publication date limit through June 1, 2026, removal of language restrictions at the search stage, eligibility of preprints, and descriptive characterization of evidence maturity instead of formal risk-of-bias assessment. These amendments and the final methods are reported transparently in Multimedia Appendix 1.

### Eligibility Criteria

#### Population

The population of interest was pediatric rare diseases and pediatric-relevant rare-disease diagnostic contexts. Studies were eligible if they enrolled children or adolescents aged 0‐18 years with a suspected or confirmed rare disease, reported pediatric data separately, focused on rare diseases with typical childhood onset or major pediatric diagnostic relevance, or evaluated diagnostic models, tools, or benchmarks whose disease spectrum included pediatric-relevant rare diseases.

We did not apply a single numerical prevalence threshold across all jurisdictions because definitions of rare disease differ between countries and may be expressed as either a prevalence rate or an absolute population count. A condition was considered eligible if the specific disease or disorder was listed in Orphanet [2], the Chinese Rare Disease Directory [11], or another official or recognized national rare-disease list applicable to the study setting. Orphanet was used as the primary reference when definitions differed across sources.

For conditions not clearly listed in these sources, eligibility required that the study explicitly identify the diagnostic target as a rare or orphan disease, Mendelian disorder, inherited metabolic disease, or condition evaluated within a recognized undiagnosed-disease or rare-disease program. When a broader condition contained both common and rare subtypes, the study was included only if the rare subtype was the specific diagnostic target and its results could be evaluated separately. Borderline or uncertain conditions were independently assessed by JZ and JL using the exact disease entity, subtype, study population, and applicable rare-disease source. Disagreements were resolved by consensus, with adjudication by YC when necessary.

Studies including both pediatric and adult populations, all-age rare-disease datasets, or benchmark datasets were retained when the diagnostic task, target disease spectrum, or intended application was directly relevant to pediatric rare-disease diagnosis. However, when pediatric-specific denominators or pediatric-specific results were not separately reported, these studies were coded and interpreted as indirect pediatric-relevant evidence rather than direct pediatric clinical validation.

#### Concept

An AI or computational model that was explicitly developed or evaluated for a diagnosis-related purpose. Eligible approaches included ML, DL, natural language processing, LLMs, facial AI, phenotype-matching systems, foundation-model architectures, and multimodal AI. Eligible tasks included rare-disease screening or cohort identification, phenotyping, diagnostic classification, differential diagnosis, phenotype or patient matching, genotype-phenotype interpretation, and variant or gene prioritization.

#### Context

Any clinical or research setting, including tertiary hospitals, registries, biobanks, retrospective databases, simulated evaluations, benchmark studies, and preprint settings.

#### Inclusion Criteria

Original studies that evaluated the diagnostic performance or utility of an eligible AI approach in a pediatric rare-disease context were included. Eligible publication types included peer-reviewed journal papers, case series, methods studies with an original diagnostic evaluation, and preprints. No language restriction was applied.

#### Exclusion Criteria

Studies were excluded if pediatric relevance could not be determined; the target condition was not rare; no eligible AI method was evaluated; or the application addressed only treatment, prognosis, monitoring, risk-factor analysis, molecular mechanism discovery, or basic science without a diagnostic application. Reviews, editorials, commentaries, letters, guidelines, protocols, conference papers or abstracts, and reports without an original diagnostic AI evaluation were excluded. For duplicate or overlapping reports, the most complete and recent version was retained.

### Information Sources and Search Strategy

The initial searches of PubMed, Scopus, and the Web of Science Core Collection were conducted on February 1, 2026, and covered publications dated from January 1, 2015, to December 31, 2025. The start date of January 1, 2015, was prespecified to ensure that early applications emerging during the contemporary machine-learning and deep-learning era were captured, rather than because eligible studies were expected to be available in every year of the search period. Because these searches were conducted after the end of 2025, studies published later in 2025 were eligible for retrieval. A final update search was conducted on June 26, 2026. PubMed, Scopus, and the Web of Science Core Collection were updated to capture publications dated through June 1, 2026, thereby also allowing records published in 2025 but indexed after the initial search to be identified. On the same date, supplementary searches were conducted in Embase, CNKI, Wanfang Data, and the Cochrane Library. Additional records were identified through medRxiv, arXiv, and hand searching of the reference lists of included studies and relevant reviews. The final study selection and analysis were based on the searches completed on June 26, 2026. No further search updates were conducted after that date. Apart from the publication-date limits, no language, age, or publication-type filters were applied at the search stage.

Search strategies generally combined controlled vocabulary and free-text terms for AI, rare diseases, pediatric populations, and diagnosis-related use. The syntax and concept-block structure were adapted to the indexing system and functionality of each source. Complete source-specific strategies, platforms, execution dates, date limits, record counts, and the rationale for the broader CNKI search are provided in Multimedia Appendix 1. The final database searches yielded 274 records from PubMed, 494 from Scopus, 201 from the Web of Science Core Collection, 1046 from Embase, 58 from CNKI, 444 from Wanfang Data, and 40 from the Cochrane Library.

### Study Selection

Records were exported to EndNote (version 21; Clarivate) and deduplicated using DOI, PMID, normalized title, publication year, and first author. Records without DOI or PMID were checked manually using title and bibliographic details. Chinese-language records were also compared with English-language records to identify translated or duplicate reports.

Screening was performed manually. JZ and JL independently screened every title and abstract in parallel using the prespecified eligibility criteria. Each reviewer recorded a decision independently, and decisions remained concealed from the other reviewer until reconciliation. Records judged potentially eligible or uncertain by either reviewer proceeded to full-text assessment. No automated ranking, stopping rule, or machine learning–assisted screening was used. Cohen κ was not computed prospectively; therefore, screening agreement was summarized using the number of initial discordant decisions and the number requiring third-reviewer adjudication at each screening stage.

JZ and JL independently assessed the full texts of potentially eligible reports. At each screening stage, every excluded record or report was assigned 1 mutually exclusive primary exclusion reason; the reason-specific counts therefore summed to the total number excluded at that stage. Disagreements were resolved through discussion and consensus, with adjudication by a third reviewer when necessary.

### Data Charting Process

A standardized data-charting form was developed in Microsoft Excel on the basis of the review question, the population-concept-context framework, and the prespecified evidence-map domains. Before final coding, the reviewers agreed on operational definitions and decision rules for the primary technology category, input modality, diagnostic task, validation strategy, publication type, and geographic assignment.

One reviewer (JZ) performed the primary data charting, after which a second reviewer (JL) independently checked every charted field against the corresponding source report for completeness and accuracy. Data charting therefore involved single-reviewer primary extraction with complete second-reviewer verification rather than 2 independent de novo extractions. Discrepancies were resolved through discussion and consensus, with adjudication by a third reviewer when necessary. Information that could not be determined from the source report was coded as not reported and was not imputed.

When a study evaluated multiple models, datasets, diagnostic tasks, or performance measures, the study was counted once in study-level summaries, and the corresponding evaluations were summarized within the same study-level entry in Table S2 of Multimedia Appendix 2 [12-92]. A distinct evaluation denominator was recorded only when it could be explicitly and unambiguously distinguished from the total study sample size; otherwise, it was coded as not reported and was not inferred. This approach preserved the relationship between the diagnostic tasks, models, evaluation datasets, reference standards, validation designs, metrics, and reported results without treating multiple evaluations from the same report as separate studies.

### Data Items and Classification Framework

Charted variables included title, first author, publication year, journal or source, publication type, country or region, study design, clinical setting, pediatric age range, sample size, rare disease or disease group, data source, input modality, AI technology and model, diagnostic task, reference standard or comparator, validation strategy, performance metrics and principal findings, and reported limitations. The study-level performance mapping summarized each study’s diagnostic task, model, evaluation dataset, reported total sample size, and unit of analysis, reference standard or comparator, validation design, performance metrics, and reported results. When a study evaluated multiple tasks, models, datasets, or metrics, the corresponding evaluations were summarized within the same study-level entry. A distinct evaluation denominator was recorded only when it was explicitly identifiable; otherwise, it was coded as not reported rather than inferred.

For descriptive evidence mapping, each study was assigned to 1 mutually exclusive primary technology category according to its principal diagnostic engine and clinical input, using the following prespecified hierarchy: multimodal AI, facial AI, LLMs, DL, and classical ML. These categories were analytical groupings developed to avoid double counting and were not intended to imply that the underlying model architectures were mutually exclusive.

Multimodal AI was assigned only when integration of 2 or more distinct data modalities within a single diagnostic model or integrated diagnostic pipeline was the central methodological feature of the study, such as joint use of imaging and clinical data or phenotypic, genomic, and metabolomic data for diagnostic inference. The mere availability of more than 1 input modality did not by itself determine assignment to the multimodal AI category. When this criterion was met, multimodal AI took precedence over LLMs, DL, and facial AI in the mutually exclusive primary-technology hierarchy. Facial AI included systems in which facial images were the principal diagnostic input and no additional distinct data modality was jointly integrated as a central methodological feature. Facial AI was retained as a separate evidence-mapping category because facial phenotyping constitutes a clinically distinct application area with specific considerations relating to image quality, age, ancestry, syndrome representation, and external validation. Facial AI systems such as DeepGestalt and GestaltMatcher were recognized as DL-based systems but were classified as facial AI for the mutually exclusive primary-technology analysis. LLMs included generative transformer-based language models used as the principal diagnostic engine in text-based or structured-vignette tasks that did not meet the multimodal AI definition. DL included other neural-network models that were neither facial AI, LLM-based, nor multimodal AI. Classical ML included nondeep supervised or unsupervised methods, statistical learning algorithms, and semantic-similarity approaches.

A foundation model was treated as an architectural descriptor rather than a separate primary technology category. A text-generative foundation model was classified as an LLM, a vision-language or other multi-input foundation model was classified as multimodal AI only when multimodal integration was the central diagnostic feature, and a nongenerative single-modality neural foundation model was classified as DL. Retrieval-augmented generation, agentic or multiagent design, phenotype-matching functionality, and underlying model architecture were retained as nonexclusive secondary descriptors. Input modalities were also coded separately and nonexclusively. Consequently, each study appeared once in the primary-technology distribution but could contribute to multiple input-modality categories. A multimodal AI study was therefore represented by 1 bar in the primary-technology figure and by each of its constituent input modalities in the input-modality figure.

Diagnostic tasks were grouped as screening or cohort identification, phenotyping or phenotype matching, diagnostic classification or differential diagnosis, variant or gene prioritization, and other diagnosis-related decision support.

Country or region was charted as a structured field for every included study. Geographic assignment was based primarily on the explicitly reported study setting or the origin of the development or validation data. When neither was reported, the country of the corresponding author’s affiliation was used; if this was unavailable, the first author’s affiliation was used. Assignments based on author affiliation were marked as inferred. For multinational studies, all represented countries were recorded, and country-level counts were therefore nonmutually exclusive. We also charted the pediatric relevance basis for each included study, distinguishing direct pediatric cohorts, mixed-age studies with pediatric relevance, and all-age or benchmark studies in which pediatric-specific results were not separately extractable.

### Critical Appraisal and Evidence Maturity

A formal risk-of-bias assessment was not undertaken because the objective of this scoping review was to map the scope, characteristics, and maturity of a heterogeneous evidence base rather than estimate a pooled effect or determine comparative effectiveness. No study was excluded or statistically weighted on the basis of methodological quality. Instead, evidence maturity was characterized descriptively according to publication status, retrospective or prospective design, single-center or multicenter data, reference standard or comparator, and the use of internal, temporal, external, or prospective validation. These dimensions were not combined into a numerical quality score but were used to contextualize the strength and clinical relevance of the reported findings. Retrospective studies with internal validation were interpreted primarily as evidence of technical feasibility; external or multicenter validation provided stronger evidence of transportability; and prospective evaluations, particularly those measuring clinician or patient outcomes, were considered more informative regarding clinical utility. Case series, proof-of-concept studies, preprints, and benchmark evaluations were interpreted as exploratory or hypothesis-generating evidence.

### Data Synthesis and Presentation

Because the included studies were heterogeneous in diseases, diagnostic tasks, datasets, reference standards, units of analysis, evaluation denominators, and outcome measures, meta-analysis was not planned. Study-level characteristics were summarized using counts and percentages, with all 81 included studies used as the denominator unless otherwise specified. Primary technology categories were mutually exclusive, whereas diagnostic tasks, input modalities, disease groups, geographic settings, and validation characteristics were nonexclusive. The denominator and nonexclusive nature of the categories were reported with the corresponding summaries.

Data synthesis was conducted in 3 stages. First, we descriptively mapped publication year, geographic setting, disease group, study design, publication type, primary AI technology, input modality, diagnostic task, and validation strategy. Second, we linked primary technology categories with validation strategies to examine whether areas with greater publication volume also demonstrated more mature evidence. Diagnostic tasks and input modalities were then examined alongside the reported performance measures to determine how the intended clinical use influenced outcome selection and interpretation. Third, reported performance was interpreted in relation to the intended diagnostic task, sample size and unit of analysis, evaluable denominator, reference standard or comparator, and validation design.

Evidence density was examined separately from evidence maturity as defined earlier. This distinction was used to identify areas in which publication activity was increasing, but external validation, prospective evaluation, or clinician-comparative assessment remained limited.

Performance measures were retained in their originally reported form. They were not pooled or used to rank AI technology categories because metrics such as sensitivity, area under the receiver operating characteristic curve (AUROC), top-k accuracy, diagnostic yield, and rank addressed different diagnostic tasks and were not directly interchangeable. Comparative statements were limited to within-study comparisons in which models or clinicians were evaluated using the same dataset, reference standard, outcome definition, and metric. No inferential or indirect statistical comparisons between technology categories were performed.

For interpretation of evidence-map density, categories represented by 1‐3 studies were classified as sparse and considered insufficient for category-level inference. Categories represented by 4‐9 studies were classified as limited and interpreted as preliminary. These thresholds were used only to describe the density of the evidence map and were not treated as methodological quality scores.

### Ethical Considerations

This review used published and publicly available information and did not involve human participants or identifiable individual-level data; therefore, institutional ethics approval was not required.

## Results

### Overview

The results are presented in 5 stages: study selection; characteristics of the included evidence; distribution of technologies, input modalities, and diagnostic tasks; validation and evidence maturity; and integrated interpretation of task-specific performance. Unless otherwise specified, percentages use all 81 included studies as the denominator. Primary technology categories were mutually exclusive, whereas disease groups, input modalities, diagnostic tasks, geographic settings, and validation characteristics were nonexclusive. Evidence density refers to the number of studies within a mapped category, whereas evidence maturity refers to the extent of external, prospective, multicenter, or clinician-comparative evaluation.

### Study Selection

The database searches identified 2557 records: PubMed (n=274), Scopus (n=494), Web of Science Core Collection (n=201), Embase (n=1046), CNKI (n=58), Wanfang Data (n=444), and the Cochrane Library (n=40). After removal of 494 duplicate records, 2063 records underwent title and abstract screening. Of these, 1957 were excluded because they did not evaluate AI for diagnosis (n=640), did not focus on a rare disease or a pediatric population (n=615), did not report diagnostic performance (n=520), or were reviews, editorials, conference abstracts, or otherwise outside the review scope (n=182). One primary exclusion reason was assigned to each excluded record; the 4 mutually exclusive categories sum to 1957. At title and abstract screening, the 2 reviewers initially disagreed on 76 of 2063 (3.7%) records; 70 disagreements were resolved through discussion, and 6 required adjudication by a third reviewer. At full-text assessment, the reviewers initially disagreed on 5 of 106 (4.7%) reports; 3 disagreements were resolved through discussion, and 2 required third-reviewer adjudication.

**Figure 1.**
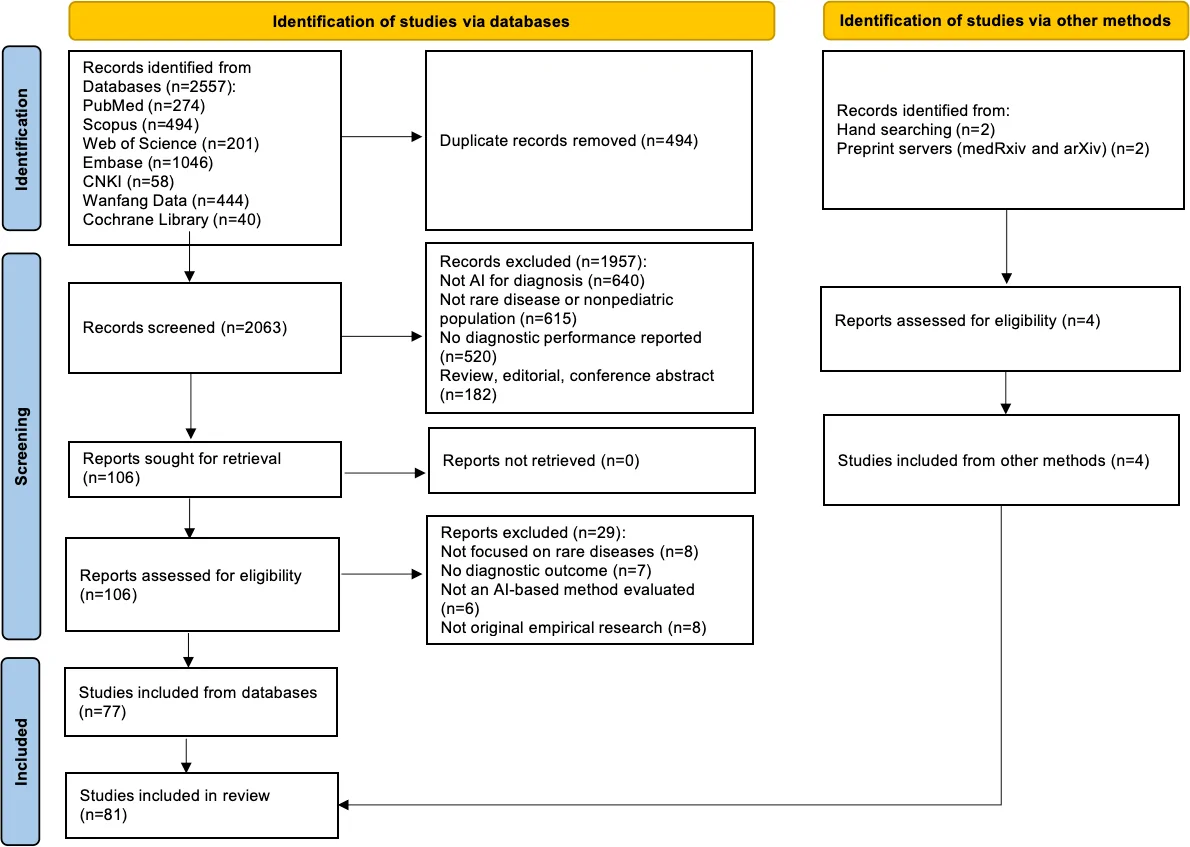
PRISMA-ScR (Preferred Reporting Items for Systematic Reviews and Meta-Analyses Extension for Scoping Reviews) flow diagram of study selection. CNKI: China National Knowledge Infrastructure.

All 106 reports sought for retrieval were obtained (reports not retrieved, n=0) and assessed in full. In total, 29 reports were excluded: 8 were not focused on rare diseases, 7 did not report a diagnostic outcome, 6 did not evaluate an eligible AI-based method, and 8 did not report original empirical research. One primary exclusion reason was assigned to each excluded report; these categories sum to 29. The full-text assessment yielded 77 studies from database searching. Four additional studies were included through hand searching (n=2) and preprint servers (medRxiv and arXiv; n=2), resulting in a final total of 81 included studies (Figure 1).

### Included Studies

The final review included 81 studies [12-92]. Their core characteristics, including publication year, country or region, target disease, study design and sample size, principal AI approach, input modality, diagnostic task, and validation strategy, are presented in Table S1 in Multimedia Appendix 2 [12-92].

### Characteristics of Included Studies

**Figure 2.**
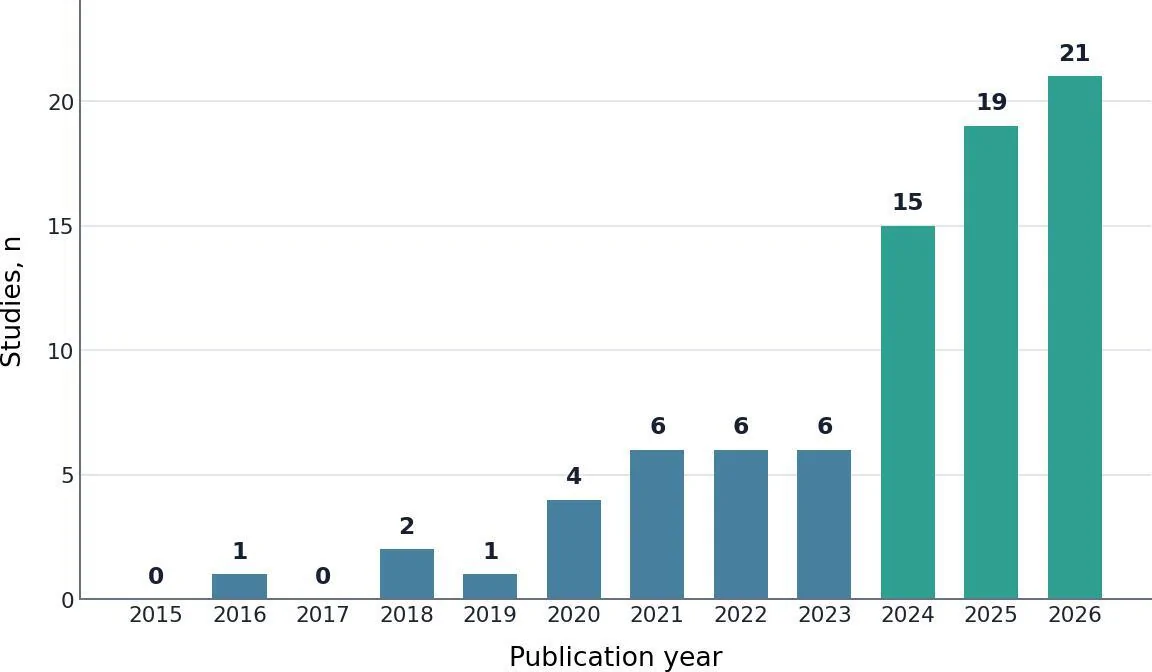
Annual publication trend of included studies. The 2026 count includes studies published through June 1, 2026.

In total, 15 of 81 (18.5%) studies were published in 2024, 19 (23.5%) in 2025, and 21 (25.9%) in 2026. Overall, 55 (67.9%) studies were published from 2024 through 2026 (Figure 2). Most reports were peer-reviewed journal papers (79/81, 97.5%); 2 (2.5%) were preprints.

**Figure 3.**
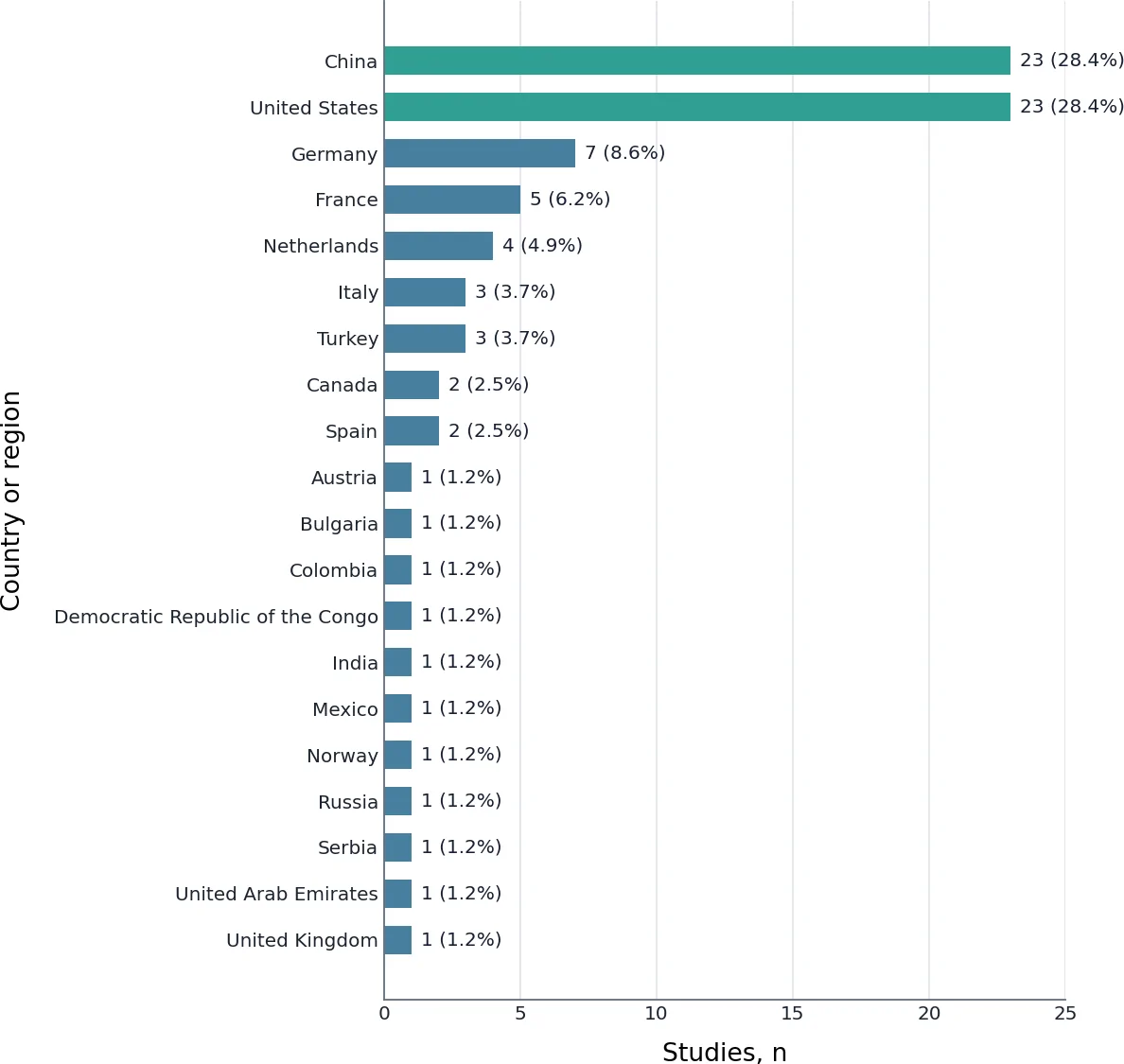
Geographic distribution of included studies by reported or inferred research setting. Country categories were nonexclusive for multinational studies because each represented country was counted. Geographic assignments based on author affiliations may not represent participant ancestry, nationality, or dataset provenance.

Based on structured geographic charting, the studies represented 20 countries, including several multinational studies. Country counts were nonmutually exclusive because every represented country was counted for multinational studies (Figure 3). China and the United States each contributed 23 of 81 (28.4%) studies, followed by Germany (7/81, 8.6%), France (5/81, 6.2%), the Netherlands (4/81, 4.9%), Turkey (3/81, 3.7%), and Italy (3/81, 3.7%). The remaining studies were distributed across Europe, Asia, North America, South America, the Middle East, and Africa. These counts describe reported or inferred research settings and should not be interpreted as the geographic or ancestral composition of the study participants or datasets.

Study-design categories were nonexclusive because some reports combined retrospective development with prospective validation. A retrospective component was present in 62 (76.5%) studies, whereas 12 (14.8%) included a prospective component. In total, 5 (6.2%) studies were cross-sectional, and 5 (6.2%) used simulation or benchmark designs. Reported sample sizes ranged from small case series to large screening datasets. A total of 2 studies did not report a sample size, and 34 did not provide numerical pediatric age information. Because the units of analysis included patients, images, variants, encounters, and case vignettes, sample sizes were not pooled.

Disease coverage was broad and fragmented. Many studies evaluated generic rare genetic, Mendelian, or multisyndromic cohorts [13,25,30,60,67,72,75,90,92], whereas others focused on a single condition or related disease group. Biliary atresia was the most frequently recurring named condition (n=4) [52,56,59,62]. Other recurring areas included inherited metabolic diseases and newborn screening [29,39,57,80,82,83,88], inborn errors of immunity and immune dysregulation [12,61,64,67,68,73], skeletal and craniofacial disorders [17,24,43,66,89], neurologic and neuromuscular conditions [36,40,84,89], retinal disorders [23], and rare syndromes with recognizable facial phenotypes [14,16,28,31-34,44-46,65,70,76,81,87]. Although biliary atresia was the most frequently recurring named condition, it was represented by only 4 studies and therefore remained a limited condition-specific evidence base. Many other named diseases or disease groups were represented by only 1‐3 studies. These sparse disease-specific cells indicate that an application has been investigated but do not support conclusions about effectiveness, generalizability, or clinical readiness for the corresponding condition.

Not all included studies provided the same level of pediatric-specific evidence. Some studies enrolled pediatric cohorts directly, whereas others used mixed-age or all-age rare-disease datasets, diagnostic tools, or benchmarks with relevance to pediatric rare-disease diagnosis. Studies without separately extractable pediatric denominators or pediatric-specific performance estimates were retained as indirect pediatric-relevant evidence and were interpreted cautiously. For example, RareArena was included as a rare-disease LLM benchmark relevant to pediatric diagnostic reasoning, but it was not treated as direct pediatric clinical validation because the full benchmark was not pediatric-dominant, and pediatric-specific results were not separately reported.

### AI Technologies and Input Modalities

**Figure 4.**
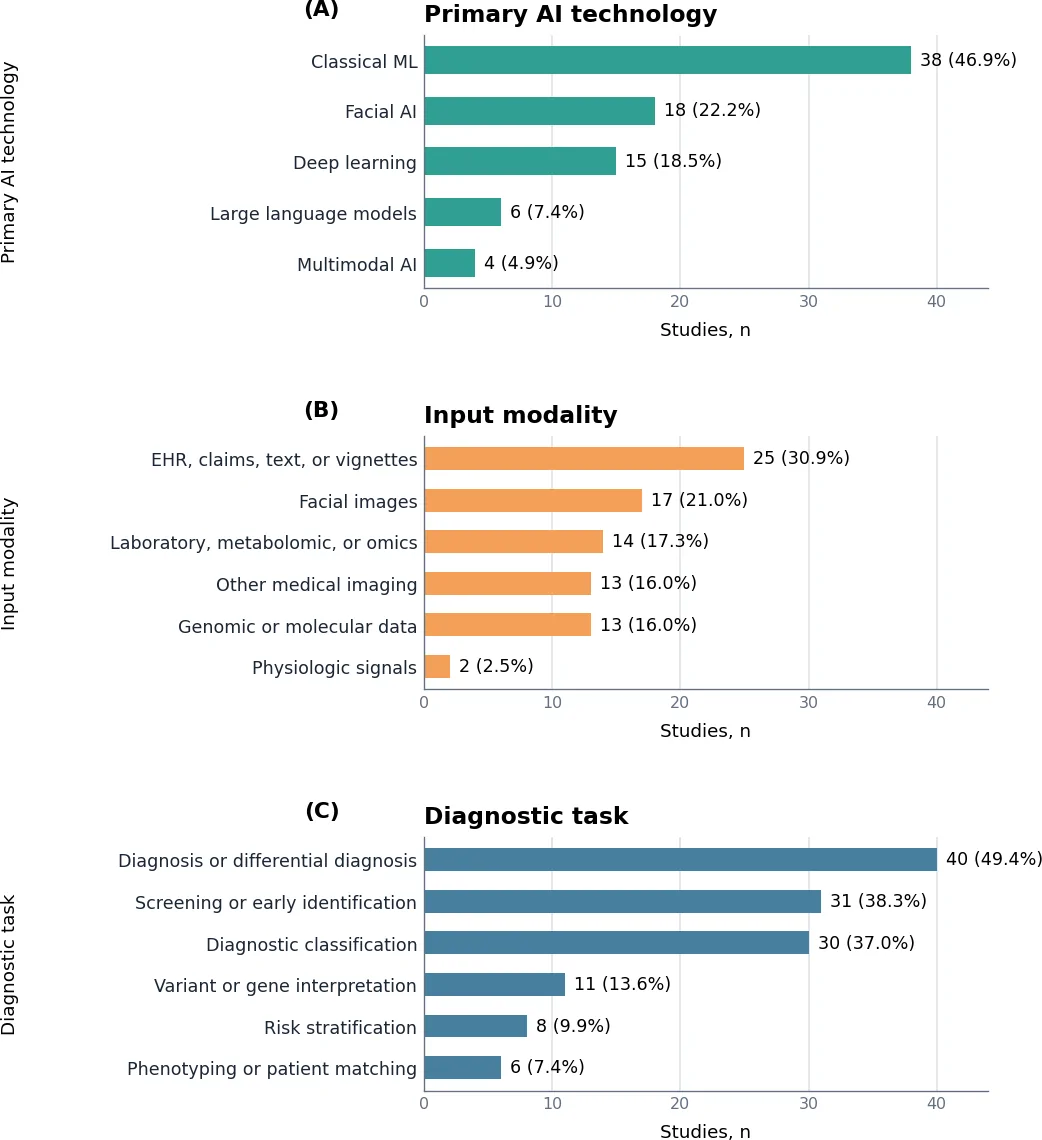
Distribution of included studies by primary AI technology, input modality, and diagnostic task. (A) Primary technology categories were mutually exclusive, and each study contributed to 1 category. Facial AI was treated as an application-level category despite frequently using deep-learning architectures. (B) Input-modality categories were nonexclusive. (C) Diagnostic-task categories were nonexclusive. Consequently, the counts in panels (B) and (C) do not sum to 81. EHR: electronic health record; ML: machine learning.

Using the mutually exclusive primary-technology hierarchy, classical ML formed the largest category (38/81, 46.9%), followed by facial AI (18/81, 22.2%), DL (15/81, 18.5%), LLMs (6/81, 7.4%; limited primary technology category), and multimodal AI (4/81, 4.9%; limited primary technology category; Figure 4A). No primary technology category met the prespecified sparse threshold of 1‐3 studies. These category sizes reflect the prespecified precedence hierarchy and were used to prevent double counting; they should not be interpreted as mutually exclusive model architectures. In particular, facial AI systems frequently used DL architectures but were grouped separately because facial images constituted their principal diagnostic input, while the DL category should be interpreted as residual deep-learning studies that were not assigned to multimodal AI, facial AI, or LLMs.

Unless otherwise specified, citations in the following descriptive results are representative examples rather than exhaustive lists; complete study-level assignments are provided in Multimedia Appendix 2. Representative studies included [12,15,18,19] for classical ML [14,16,28], for facial AI [13,17,23], for DL [36,59,80,91], and for multimodal AI, while the 6 LLM studies were [21,49,51,78,86,90]. Classical ML approaches included random forests, support vector machines, gradient-boosting methods, logistic regression, Bayesian models, and semantic-similarity algorithms. DL studies used convolutional, graph-based, and transformer architectures across clinical, imaging, and molecular data. Foundation-model architecture, retrieval augmentation, agentic or multiagent design, phenotype-matching functionality, and underlying model architecture were coded as nonexclusive secondary descriptors.

Input-modality categories were nonexclusive. Electronic health records, claims, clinical text, or structured clinical vignettes were used in 25 (30.9%) studies (representative studies [18-22,26,34,35,37,38,42,47,49,50,54,55,60,68,71]). Facial images were used in 17 (21%) studies [14,16,28,31-34,41,44-46,65,70,72,76,81,87], and other medical imaging was used in 13 (16%) studies [17,23,24,27,43,52,53,59,62,66,77,89,91]. Genomic or molecular data were used in 13 (16%) studies [13,15,25,30,40,63,64,67,72,75,80,90,92], while laboratory, metabolomic, or other omics data were used in 14 (17.3%) studies (representative studies [12,29,39,52,56,57,59,61,69,80,82,83,88]). Physiologic signals were used in 2 (2.5%) studies [36,48]. In total, 4 studies were assigned to the primary multimodal AI category [36,59,80,91], and several studies assigned to other primary technology categories also used more than 1 input modality (Figure 4B).

### Diagnostic Tasks

Diagnostic-task categories were also nonexclusive. Diagnosis or differential diagnosis was evaluated in 40 of 81 (49.4%) studies (representative studies [49,51,60,90]), screening or early identification in 31 (38.3%; representative studies [18,22,47,65,80]), and diagnostic classification in 30 (37%; representative studies [14,35,73,89]). Less frequently studied tasks included risk stratification (8/81, 9.9%; representative studies [40,50,68,69]), variant or gene interpretation (11/81, 13.6%; representative studies [13-15,30,63,64,75,92]), and phenotyping or patient matching (6/81, 7.4%; studies [13,33,37,38,41,45]; Figure 4C). Risk stratification and phenotyping or patient matching were considered limited task categories. Automated bone-age assessment [66] and hormone prediction [91] were sparse task categories and were mapped descriptively without category-level inference.

The relationship between diagnostic task, input data, and reported outcome measures is summarized in Table 1. LLM studies were concentrated in differential diagnosis, rare-disease screening, and diagnostic confirmation using clinical narratives or structured case vignettes.

Because the meaning of a performance metric depends on the diagnostic task, Table 1 summarizes the principal AI approaches, input data, and commonly reported outcomes within each task category. Task categories were nonexclusive, and the table is intended to support interpretation rather than direct comparison or ranking of technologies.

**Table 1.** Descriptive summary of AI approaches and reported outcomes by diagnostic task.^a^

Diagnostic task	Common AI approaches and input data	Commonly reported outcomes	Interpretation
Screening or early identification (n=31) [18,21,22,47,65,80]	Predominantly classical ML^b^ and DL^c^, with selected multimodal AI and LLM^d^ evaluations; EHRs^e^, claims, laboratory results, metabolomic data, imaging, and clinical narratives	Sensitivity, specificity, AUROC^f^, PPV^g^, NPV^h^, calibration, false-positive rate, and diagnostic yield	Evaluates whether potentially affected children can be identified before definitive diagnosis. Results depend strongly on disease prevalence, decision thresholds, and the clinical spectrum of the screened population.
Diagnosis or differential diagnosis (n=40) [21,49,51,60,78,86,90]	Classical ML, LLMs, retrieval-augmented or agentic systems, and multimodal AI; clinical narratives, structured vignettes, phenotypes, genomic findings, and confirmatory test results	Top-k accuracy or recall, rank of the correct diagnosis, diagnostic accuracy, and clinician or expert comparison	Performance depends on candidate-set size, case difficulty, information completeness, prompt design, and whether confirmatory test results are provided.
Diagnostic classification (n=30) [14,35,73,89]	Facial AI, classical ML, and DL; facial images, other medical imaging, laboratory data, and structured clinical variables	Accuracy, AUROC, sensitivity, specificity, precision, and *F*_1_- score	Usually evaluates classification within a predefined disease set. High performance in a restricted classifier should not be interpreted as performance in an unrestricted rare-disease differential diagnosis.
Risk stratification (n=8) [40,50,68,69]	Primarily classical ML using EHR, demographic, clinical, and laboratory variables	AUROC, sensitivity, specificity, PPV, NPV, and risk-group classification	Identifies patients at increased diagnostic risk but does not necessarily establish the final diagnosis.
Variant or gene interpretation (n=11) [13-15,30,63,64,75,92]	Classical ML, DL, phenotype-genotype matching, and selected multimodal AI models; genomic variants, gene-level features, and standardized phenotypes	Top-k gene or variant recall, rank of the causal gene or variant, and diagnostic yield	Results depend on the molecular reference standard, candidate-variant filtering, phenotype completeness, and whether the causal variant was represented in the candidate set.
Phenotyping or patient matching (n=6) [13,33,37,38,41,45]	Facial AI, semantic-similarity methods, and DL; facial images, Human Phenotype Ontology terms, and structured phenotypic profiles	Top-k match rate, similarity score, rank, recall, and AUROC	Performance may be influenced by database composition, ancestry, age, image quality, phenotype coding, and representation of the target syndrome.

a Diagnostic-task categories were nonexclusive; therefore, study counts do not sum to 81. The listed outcomes reflect measures commonly reported within each task and should not be interpreted as directly comparable across rows or technology categories. Task categories represented by 1‐3 studies were considered sparse and insufficient for category-level inference, whereas categories represented by 4‐9 studies were considered limited and interpreted as preliminary. Automated bone-age assessment and hormone prediction were each represented by 1 study and were mapped descriptively only. Citations shown in this table are representative examples rather than exhaustive lists; complete study-level assignments are provided in Multimedia Appendix 2.

b ML: machine learning.

c DL: deep learning.

d LLM: large language model.

e EHR: electronic health record.

f AUROC: area under the receiver operating characteristic curve.

g PPV: positive predictive value.

h NPV: negative predictive value.

### Validation Strategies and Evidence Maturity

**Figure 5.**
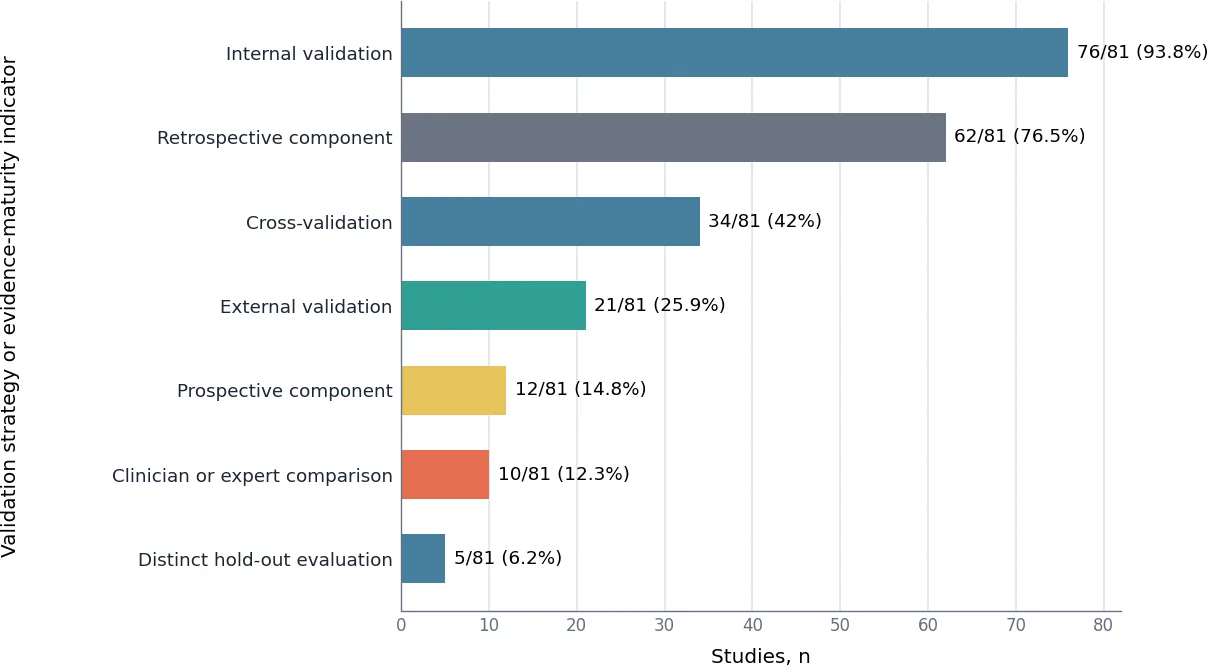
Validation strategies and evidence-maturity indicators among included studies. Categories were nonexclusive because individual studies could have more than 1 study design or validation characteristic. Cross-validation and distinct hold-out evaluation were treated as subsets of internal validation.

Evidence maturity varied substantially across the included studies. Validation strategies were nonmutually exclusive. Internal validation was explicitly reported in 76 (93.8%) studies, including cross-validation in 34 (42%) and a distinct hold-out evaluation in 5 (6.2%). External validation was reported in 21 (25.9%) studies (representative studies [13,20,52,59,62,66,71,90]), 12 (14.8%) studies included a prospective component (representative studies [14,47,49,53,59,72]), and 10 (12.3%) studies compared model outputs with clinicians or domain experts (representative studies [25,31,44,49,60,70]). Together with the predominance of retrospective designs reported earlier, these findings indicate that the evidence base remained dominated by model development and internal evaluation, with a smaller body of externally validated, prospective, or clinician-comparative evidence (Figure 5).

### Task-Specific Performance Findings

Because the included studies differed substantially in diagnostic task, unit of analysis, validation design, reference standard, and reporting of evaluable denominators, performance findings were summarized descriptively rather than compared quantitatively. In many studies, the total development or study sample size was reported, but the denominator corresponding to a specific performance metric was not separately available. Therefore, study-level task, sample size, validation design, metric, reported result, and reported evaluation set or denominator are provided in Table S2 in Multimedia Appendix 2.

Outcome reporting was heterogeneous and was therefore interpreted according to the corresponding diagnostic task rather than the technology category alone. Accuracy was reported in 34 (42%) studies, sensitivity or specificity in 26 (32.1%), area under the curve or AUROC in 25 (30.9%), top-k accuracy, recall, or rank in 25 (30.9%), *F*_1_-score in 14 (17.3%), and positive or negative predictive value in 10 (12.3%). These metrics addressed different clinical questions: screening studies commonly emphasized sensitivity and predictive values, differential-diagnosis studies used top-k accuracy or rank, and restricted classification studies generally reported accuracy or AUROC.

Because the target diseases, diagnostic tasks, class distributions, reference standards, units of analysis, evaluable denominators, and validation designs differed substantially, performance estimates were not pooled or used for direct comparisons between technology categories. Broad cross-study performance intervals were removed from the narrative synthesis. Study-level mappings of diagnostic task, reported total sample size and unit of analysis, explicitly identifiable evaluation denominator where available, validation design, metric, and reported result are provided in Table S2 of Multimedia Appendix 2. The examples presented below should therefore be interpreted only within their original study contexts.

Several condition-specific models reported strong discrimination, although their clinical maturity differed. Externally evaluated biliary-atresia models reported high performance on independent datasets [52,59,62]. A multimodal AI newborn-screening study reported high sensitivity and disease-specific reductions in false-positive results [80], whereas an electroencephalography-electromyography fusion model reported high accuracy for infantile epileptic spasms syndrome [36]. Facial AI studies frequently reported high area under the curve or accuracy for selected syndromes [31,32,44-46,65,81], but performance varied across syndromes and populations, and only a subset used external datasets or direct clinician comparisons [33,46,65,70].

In total, 6 studies evaluated LLMs in pediatric rare-disease diagnosis [21,49,51,78,86,90]. The reported findings varied substantially according to the diagnostic task and information provided. In rare pediatric case reports, top-1 diagnostic accuracy ranged from 8.2% to 13.1% [86]. In a skeletal-dysplasia benchmark, ChatGPT and DeepSeek achieved top-3 accuracies of 62.2% and 64.4%, respectively, compared with 82.2% for a clinical expert panel [49]. In RareArena, GPT-4o achieved a top-1 recall of 33.1% for rare-disease screening and 64.2% for diagnostic confirmation when test results were available [21]. Retrieval-augmented [51] and agentic systems [78,90] reported improvements in selected evaluations. However, these studies differed substantially in their case construction, diagnostic tasks, available information, model versions, prompting strategies, retrieval components, and outcome definitions. The findings should therefore be regarded as preliminary, study-specific observations rather than evidence of the overall diagnostic capability of LLMs.

### Integrated Evidence Synthesis

Linking the primary technology categories with validation strategies showed that evidence density and evidence maturity did not progress in parallel. Among the 38 classical ML studies, 7 included external validation, and 2 included a prospective component. Among the 18 facial AI studies, 3 included external validation, and 5 included a prospective component. Of the 15 DL studies, 8 included external validation, and 3 included a prospective component. Among the 6 LLM studies, 2 included external validation and 1 included a prospective component, while 1 of the 4 multimodal AI studies included external validation and 1 included a prospective component. Validation characteristics were nonexclusive, and an individual study could contribute to more than 1 evidence-maturity indicator. Because the LLM and multimodal AI categories had small denominators, these distributions should be interpreted as evidence-mapping signals rather than comparisons of maturity or effectiveness between technologies.

Across the evidence map, publication growth primarily reflected continued model development rather than a corresponding increase in evidence of clinical utility. Although 55 of 81 (67.9%) studies were published from 2024 through 2026, 62 (76.5%) included a retrospective component and 76 (93.8%) reported internal validation. In contrast, 21 (25.9%) studies included external validation, 12 (14.8%) included a prospective component, and 10 (12.3%) compared model outputs with clinicians or domain experts. These findings indicate that most of the current literature supports technical feasibility, whereas evidence for transportability across settings and incremental clinical utility remains comparatively limited.

The linked task-based synthesis also showed that performance measures reflected different clinical questions. Screening studies generally emphasized sensitivity, specificity, predictive values, and false-positive burden; differential-diagnosis studies commonly used top-k recall or the rank of the correct diagnosis; restricted classification studies primarily reported accuracy or AUROC; and variant- or gene-prioritization studies used causal-gene rank or diagnostic yield. These outcomes were not interchangeable. Consequently, favorable performance within a restricted classifier or curated benchmark should not be interpreted as evidence of superior performance in an unrestricted clinical diagnostic workflow.

## Discussion

### Principal Findings

The central finding of this review is a mismatch between the breadth of research activity and the maturity of the supporting evidence. Most studies remained retrospective and relied primarily on internal validation, whereas externally validated, prospective, and clinician-comparative evaluations constituted a smaller part of the evidence base. Favorable discrimination, classification, or ranking performance should therefore be interpreted as evidence of technical promise rather than established clinical effectiveness.

Pediatric rare-disease AI is not a single diagnostic problem. Screening, differential diagnosis, diagnostic classification, phenotype matching, and molecular interpretation require different inputs, reference standards, outcome measures, and performance priorities. The evidence map should consequently be interpreted as a description of research activity and maturity rather than a hierarchy of technologies. Retrospective internally validated studies primarily establish technical feasibility; external or multicenter validation provides stronger evidence of transportability; and prospective evaluations are needed to establish clinical utility. Case series, proof-of-concept studies, preprints, and benchmark evaluations remain exploratory. Cross-category performance summaries are therefore descriptive and should not be used to rank AI approaches. Table 1 provides a task-based guide to explain why different studies selected different performance measures; it is not intended to support indirect comparisons between classical ML, facial AI, multimodal AI, and LLMs. Evidence density was uneven across the map. Physiologic-signal inputs (n=2), automated bone-age assessment (n=1), hormone prediction (n=1), and many individual disease-specific applications were supported by only 1‐3 studies and were therefore too sparse for category-level inference. Multimodal AI (n=4), LLMs (n=6), phenotyping or patient matching (n=6), and risk stratification (n=8) were also represented by limited numbers of studies and should be interpreted as preliminary. Within the LLM and multimodal AI categories, several individual architectures or system configurations were evaluated in only 1 or 2 studies. These cells establish the presence of research activity but cannot determine comparative effectiveness, generalizability, or clinical readiness.

### Comparison With Previous Research

Previous reviews generally considered classical ML, DL, or LLMs separately [4-7]. The final search for the present review covered 7 bibliographic databases, 2 preprint servers, and reference-list searching. Adding Embase, the Cochrane Library, CNKI, and Wanfang Data broadened the search beyond the 3 English-language databases used initially and increased the potential identification of Chinese-language and regionally published studies. Nevertheless, the included evidence remained concentrated in China, the United States, and a small number of European countries and largely originated from tertiary or research-intensive settings. Database expansion therefore improved search coverage but should not be interpreted as establishing comprehensive geographic or linguistic representativeness.

Earlier reviews characterized LLM applications in pediatric rare diseases as sparse [4,7]. Although the updated review identified 6 relevant studies [21,49,51,78,86,90], the evidence remains too limited and heterogeneous to support general conclusions about LLM capabilities. Differences in case formulation, information completeness, model configuration, prompting or retrieval strategy, and outcome definition make the available findings preliminary and study-specific. Standardized, independently developed, and clinically representative evaluations are required before broader conclusions can be drawn.

### Clinical and Methodological Implications

Evaluation should be organized around the intended clinical decision. Screening models should prioritize sensitivity, negative predictive value, calibration, and the workload generated by false-positive alerts. Differential-diagnosis systems should report top-k recall, rank of the correct diagnosis, calibration, and performance on diagnostically difficult cases rather than top-1 accuracy alone. Confirmation systems should demonstrate how laboratory, imaging, pathological, or genomic evidence supports the final diagnosis. Variant- and gene-prioritization tools should report rank-based performance and diagnostic yield using an appropriate molecular reference standard.

Future evaluations should separate model development from a genuinely independent test phase. Temporal, geographic, and center-based splits are preferable to random internal splits when the intended claim concerns generalizability. External validation should preserve the target prevalence and clinical spectrum, including incomplete phenotypes and common mimics. For tools intended for clinical decision support, comparison should move beyond model versus clinician accuracy toward clinician-alone versus clinician-plus-AI designs, with measurement of diagnostic time, test use, referral appropriateness, diagnostic yield, and potential patient harm.

### LLMs and Multimodal AI

LLMs offer a practical interface for synthesizing longitudinal clinical narratives and generating differential diagnoses, but their outputs may be sensitive to prompt wording, case structure, information completeness, model version, and decoding settings. Benchmark results may also vary according to case source, disease spectrum, candidate-list size, availability of confirmatory test results, and the selected evaluation metric. Consequently, performance observed in a curated benchmark may not transfer directly to real pediatric records, in which phenotypes are often incomplete, longitudinal, age-dependent, inconsistently documented, or explicitly negated. Several included studies used structured phenotype representations, including Human Phenotype Ontology (HPO)–based information [13,37,38,51,90]. Such representations may help standardize clinical inputs and connect LLMs with phenotype and genomic knowledge resources. However, automated phenotype extraction may introduce errors through missed findings, incorrect negation handling, loss of temporal context, or failure to recognize developmentally changing phenotypes. Future studies should therefore compare raw clinical text, automatically extracted HPO terms, and clinician-curated phenotype representations and should report prompts, model versions, decoding settings, and benchmark construction transparently. Retrieval-augmented and agentic workflows may improve traceability, but their incremental clinical value still requires controlled, reproducible, and prospective evaluation.

Multimodal AI approaches are clinically well aligned with pediatric rare-disease diagnosis because they can integrate phenotypic, imaging, laboratory, and genomic evidence. However, the available evidence is too sparse to determine whether multimodal AI systems provide consistent incremental value over single-modality approaches. Their apparent performance may also be influenced by data leakage, selective availability of confirmatory tests, or evaluation after the diagnostic pathway is already complete. Future studies should use controlled ablation analyses, independent external datasets, and prospective workflows and should clearly distinguish information available during early screening from evidence obtained during diagnostic confirmation.

### Equity and Implementation Considerations

Despite the expanded database coverage, the evidence remained concentrated in China, the United States, and a small number of European countries. Few studies were conducted in low-resource settings, and population diversity was inconsistently reported. Geographic concentration may limit the transportability of diagnostic AI because disease prevalence, genetic ancestry, phenotype documentation, language, health information systems, referral pathways, and access to confirmatory testing differ across settings. These concerns are particularly relevant to facial, genomic, and language-based models, whose performance may be sensitive to ancestry, data representation, language, and local diagnostic practice. Future studies should therefore prioritize external validation in underrepresented populations and settings and report subgroup performance rather than assuming that findings from a single country or tertiary center are broadly generalizable.

Implementation also requires attention to privacy, consent, data governance, explainability, and accountability for false reassurance or unnecessary testing. The inclusion of preprints improved the timeliness of the evidence map but also meant that some emerging findings had not undergone full peer review. Clinical adoption should depend on transparent documentation, version control, postdeployment monitoring, and a clear definition of the clinician’s role in reviewing model outputs.

### Limitations

This review has several limitations. First, although the review was registered in PROSPERO before study screening, data charting, and evidence synthesis were completed, registration occurred after the initial searches conducted on February 1, 2026. In addition, the original publicly available version 1.0 record described the initial 3-database strategy and did not describe the subsequent methodological expansion to the final search of 7 bibliographic databases, 2 preprint servers, and reference-list hand searching. This expansion was undertaken during paper revision to improve search coverage and reproducibility, and the amended PROSPERO record was publicly updated as version 1.1 on July 27, 2026. Second, despite searching 7 bibliographic databases, 2 preprint servers, and reference lists, relevant studies may have been missed because terminology for AI and rare diseases is evolving rapidly. Third, disease definitions, pediatric age reporting, study units, reference standards, and performance metrics were highly heterogeneous; 34 studies did not provide numerical pediatric age information, and 2 did not report sample size. In addition, a distinct evaluation denominator could not be consistently separated from the total study sample size in the charted information. Evaluation denominators were therefore reported only when explicitly identifiable and were not inferred. Fourth, technology, modality, disease, and task categories required reviewer judgment. Input modalities and diagnostic tasks were intentionally nonexclusive, so their counts should not be summed to obtain the total number of studies. Country or region could not always be determined directly from the reported study setting or the origin of the development and validation data. When this information was unavailable, geographic assignment was inferred from the corresponding author’s affiliation or, if unavailable, the first author’s affiliation. Consequently, the geographic distribution reported in this review represents reported or inferred research settings and may not reflect participants’ nationality or ancestry or the geographic provenance of the underlying datasets.

Fifth, no formal risk-of-bias assessment was undertaken, consistent with the mapping purpose of this scoping review. Reported performance therefore describes the published evidence but should not be interpreted as a comparative ranking of technologies or as proof of clinical effectiveness. Sixth, inclusion of 2 preprints improved coverage of emerging work but introduced evidence that had not undergone full peer review. Finally, the search was limited to publications dated through June 1, 2026; given the pace of model development, the LLM and multimodal AI sections will require periodic updating.

### Recommendations for Future Research

Future research could prioritize prospective multicenter studies, independent external validation, and standardized task-specific reporting. Reproducible evaluations should prespecify eligibility criteria, reference standards, data-freezing rules, model names and versions, access dates, system and user prompts, decoding parameters, repeated-run procedures, retrieval sources, phenotype-extraction methods and HPO versions, and locked test sets. Reporting should include pediatric age distribution, disease prevalence, class balance, missing-data handling, subgroup performance, calibration, uncertainty, and clinically relevant error analysis. Shared benchmarks should distinguish early rare-disease screening from diagnosis after confirmatory test results become available.

Research should also evaluate workflow and patient-centered outcomes, including time to diagnosis, diagnostic yield, unnecessary testing, referral patterns, clinician trust, family understanding, equity, and cost. Human-in-the-loop studies are needed to establish when AI improves expert reasoning, when it creates automation bias, and which explanations or evidence displays support safe use at the point of care.

### Conclusions

AI approaches now address multiple diagnosis-related tasks in pediatric rare diseases, but the heterogeneous and predominantly retrospective evidence does not establish generalizable clinical benefit. This evidence map identified gaps in independent external validation, prospective evaluation, reproducible task-specific assessment, and measurement of the incremental value of AI to clinicians. Future research could address these gaps while distinguishing early rare-disease screening from evidence-rich diagnostic confirmation.

## Supplementary material

10.2196/96169 Multimedia Appendix 1 Full search strategy.

10.2196/96169 Multimedia Appendix 2 Core characteristics and study-level performance mapping.

10.2196/96169 Checklist 1 PRISMA-ScR checklist.

## Abbreviations

AUROC: area under the receiver operating characteristic curve
CNKI: China National Knowledge Infrastructure
DL: deep learning
EHR: electronic health record
HPO: Human Phenotype Ontology
LLM: large language model
ML: machine learning
PRISMA-ScR: Preferred Reporting Items for Systematic Reviews and Meta-Analyses Extension for Scoping Reviews
PROSPERO: International Prospective Register of Systematic Reviews
