# Supplementary material for: Machine Learning, Large Language Models, and Multimodal AI for Diagnosing Pediatric Rare Diseases: Scoping Review
Source: J Med Internet Res. 2026 Aug 28;28:e96169. doi: 10.2196/96169 (PMC13524366; doi:10.2196/96169)
Supplement: Supplementary file 1 [file jmir-v28-e96169-s001.docx]

**Search Overview**

The revised scoping review search was designed to identify studies evaluating artificial intelligence methods for diagnostic use in pediatric rare diseases. The original search covered PubMed, Scopus, and Web of Science from 1 January 2015 to 31 December 2025. For this revision, all database searches were rerun on 26 June 2026 using revised database-specific strategies and a publication date range of 1 January 2015 to 1 June 2026. Coverage was expanded to Embase, the Cochrane Library, China National Knowledge Infrastructure (CNKI), and Wanfang Data. Hand searching and searches of medRxiv and arXiv were also conducted as additional identification methods.

Search strategies generally covered artificial intelligence, rare diseases, pediatric populations, and diagnosis-related use. Search syntax and concept-block structure were adapted to the indexing system and functionality of each source. For CNKI, a broader 2-block strategy covering artificial intelligence and rare diseases was used to maximize sensitivity; pediatric and diagnostic relevance were subsequently assessed during screening.

No language, age, or publication-type filters were applied at the search stage unless required by the database interface. Pediatric, rare disease, artificial intelligence, and diagnostic concepts were included directly in the search strings. Chinese and English records were eligible if sufficient information was available for screening and data extraction. Search strings are reported in database-specific syntax below; line breaks were added for readability.

The database searches identified 2557 records: PubMed (n=274), Scopus (n=494), Web of Science Core Collection (n=201), Embase (n=1046), CNKI (n=58), Wanfang Data (n=444), and the Cochrane Library (n=40). After removing 494 duplicate records, 2063 records underwent title and abstract screening and 1957 were excluded. Of 106 reports assessed in full text, 29 were excluded and 77 studies from databases were included. Four additional records were identified through hand searching (n=2) and preprint servers (medRxiv and arXiv; n=2); all four were assessed and included. The final review comprised 81 studies.

**Sources, Platforms, Limits, and Records Retrieved**

| **Source** | **Platform/interface** | **Date range and limits** | **Records identified** |
| --- | --- | --- | --- |
| PubMed | PubMed, National Library of Medicine | 1 Jan 2015 to 1 Jun 2026; search run 26 Jun 2026; no language, age, or publication-type restriction. | n=274 |
| Scopus | Scopus, Elsevier | 1 Jan 2015 to 1 Jun 2026; search run 26 Jun 2026; no language, age, or document-type restriction. | n=494 |
| Web of Science | Web of Science Core Collection, Clarivate | 1 Jan 2015 to 1 Jun 2026; search run 26 Jun 2026; no language, age, or document-type restriction. | n=201 |
| Embase | Embase.com, Elsevier | 1 Jan 2015 to 1 Jun 2026; search run 26 Jun 2026; no language, age, or document-type restriction. | n=1046 |
| CNKI | China National Knowledge Infrastructure | 1 Jan 2015 to 1 Jun 2026; search run 26 Jun 2026; Chinese and English records eligible. | n=58 |
| Wanfang Data | Wanfang Data Knowledge Service Platform | 1 Jan 2015 to 1 Jun 2026; search run 26 Jun 2026; Chinese and English records eligible. | n=444 |
| Cochrane Library | Cochrane Library, Wiley | 1 Jan 2015 to 1 Jun 2026; search run 26 Jun 2026; no language restriction. | n=40 |
| Hand searching | Reference lists of included reports and relevant prior reviews | Completed 26 Jun 2026. | n=2 |
| Preprint servers | medRxiv and arXiv | 1 Jan 2015 to 1 Jun 2026; search run 26 Jun 2026. | n=2 |

**Protocol amendment note**

The original PROSPERO Version 1.0 record described the initial 3-database scoping review. During manuscript revision, the search strategy was expanded to improve coverage and reproducibility. The final review searched 7 bibliographic databases, including PubMed, Scopus, Web of Science Core Collection, Embase, CNKI, Wanfang Data, and the Cochrane Library, supplemented by medRxiv, arXiv, and reference-list hand searching. The final database searches were conducted on June 26, 2026, and covered records published from January 1, 2015, to June 1, 2026. The amended PROSPERO record was publicly updated as Version 1.1 on July 27, 2026.

**Database-Specific Search Strategies**

**PubMed (n=274)**

Platform: PubMed, National Library of Medicine. Search date: 26 June 2026. Publication date range: 1 January 2015 to 1 June 2026.

#1 Artificial intelligence block

("Artificial Intelligence"[Mesh] OR "Machine Learning"[Mesh] OR "Deep Learning"[Mesh] OR "Natural Language Processing"[Mesh] OR "Algorithms"[Mesh] OR "artificial intelligence"[Title/Abstract] OR "AI"[Title/Abstract] OR "machine learning"[Title/Abstract] OR "deep learning"[Title/Abstract] OR "large language model*"[Title/Abstract] OR "LLM"[Title/Abstract] OR "LLMs"[Title/Abstract] OR "ChatGPT"[Title/Abstract] OR "GPT-4"[Title/Abstract] OR "GPT"[Title/Abstract] OR "foundation model*"[Title/Abstract] OR "generative AI"[Title/Abstract] OR "multimodal AI"[Title/Abstract] OR "multimodal model*"[Title/Abstract] OR "vision-language model*"[Title/Abstract] OR "natural language processing"[Title/Abstract] OR "facial recognition"[Title/Abstract] OR "face analysis"[Title/Abstract] OR "phenotypic matching"[Title/Abstract] OR algorithm*[Title/Abstract])

#2 Rare disease block

("Rare Diseases"[Mesh] OR "rare disease*"[Title/Abstract] OR "orphan disease*"[Title/Abstract] OR "rare genetic disease*"[Title/Abstract] OR "rare genetic disorder*"[Title/Abstract] OR "Mendelian disease*"[Title/Abstract] OR "Mendelian disorder*"[Title/Abstract] OR "undiagnosed disease*"[Title/Abstract])

#3 Pediatric block

("Pediatrics"[Mesh] OR "Child"[Mesh] OR "Infant"[Mesh] OR "Adolescent"[Mesh] OR pediatric*[Title/Abstract] OR paediatric*[Title/Abstract] OR child*[Title/Abstract] OR infant*[Title/Abstract] OR newborn*[Title/Abstract] OR neonat*[Title/Abstract] OR adolescent*[Title/Abstract] OR youth*[Title/Abstract])

#4 Diagnosis block

("Diagnosis"[Mesh] OR diagnos*[Title/Abstract] OR "differential diagnosis"[Title/Abstract] OR "diagnostic accuracy"[Title/Abstract] OR screening[Title/Abstract] OR "case classification"[Title/Abstract] OR "variant prioritization"[Title/Abstract] OR "variant prioritisation"[Title/Abstract] OR phenotyp*[Title/Abstract] OR "phenotype matching"[Title/Abstract])

#5 Final PubMed search

#1 AND #2 AND #3 AND #4 AND ("2015/01/01"[Date - Publication] : "2026/06/01"[Date - Publication])

**Embase (n=1046)**

Platform: Embase.com, Elsevier. Search date: 26 June 2026. Publication date range: 1 January 2015 to 1 June 2026.

The following strategy is reported in Embase.com syntax. No language, age, or document-type restrictions were applied at the search stage.

#1 Artificial intelligence block, focused

('artificial intelligence':ti,ab,kw OR 'machine learning':ti,ab,kw OR 'deep learning':ti,ab,kw OR 'neural network*':ti,ab,kw OR 'random forest*':ti,ab,kw OR 'support vector machine*':ti,ab,kw OR xgboost:ti,ab,kw OR 'large language model*':ti,ab,kw OR llm*:ti,ab,kw OR chatgpt:ti,ab,kw OR 'gpt-4':ti,ab,kw OR 'gpt-3.5':ti,ab,kw OR 'generative pre-trained transformer*':ti,ab,kw OR 'foundation model*':ti,ab,kw OR 'generative ai':ti,ab,kw OR 'multimodal ai':ti,ab,kw OR 'multimodal model*':ti,ab,kw OR 'vision-language model*':ti,ab,kw OR 'vision language model*':ti,ab,kw OR 'natural language processing':ti,ab,kw OR 'facial recognition':ti,ab,kw OR 'facial analysis':ti,ab,kw OR 'face analysis':ti,ab,kw OR 'phenotype matching':ti,ab,kw OR 'phenotypic matching':ti,ab,kw)

#2 Rare disease block

('rare disease'/exp OR 'rare disease*':ti,ab,kw OR 'orphan disease*':ti,ab,kw OR 'rare genetic disease*':ti,ab,kw OR 'rare genetic disorder*':ti,ab,kw OR 'mendelian disease*':ti,ab,kw OR 'mendelian disorder*':ti,ab,kw OR 'undiagnosed disease*':ti,ab,kw)

#3 Pediatric block, text-field only

(pediatric*:ti,ab,kw OR paediatric*:ti,ab,kw OR child*:ti,ab,kw OR childhood:ti,ab,kw OR infant*:ti,ab,kw OR newborn*:ti,ab,kw OR neonat*:ti,ab,kw OR adolescent*:ti,ab,kw OR youth*:ti,ab,kw)

#4 Diagnostic-use block, focused

(diagnos*:ti,ab,kw OR 'differential diagnosis':ti,ab,kw OR 'diagnostic accuracy':ti,ab,kw OR screening:ti,ab,kw OR 'newborn screening':ti,ab,kw OR 'case classification':ti,ab,kw OR 'clinical decision support':ti,ab,kw OR 'variant prioritization':ti,ab,kw OR 'variant prioritisation':ti,ab,kw OR 'phenotype matching':ti,ab,kw OR 'phenotypic matching':ti,ab,kw)

#5 Final Embase search

#1 AND #2 AND #3 AND #4

Limits: publication date 1 January 2015 to 1 June 2026; no language or document-type restriction.

**Scopus (n=494)**

Platform: Scopus, Elsevier. Search date: 26 June 2026. Publication date range: 1 January 2015 to 1 June 2026.

TITLE-ABS-KEY("artificial intelligence" OR "machine learning" OR "deep learning" OR "large language model*" OR llm OR llms OR chatgpt OR "gpt-4" OR gpt OR "foundation model*" OR "generative ai" OR "multimodal ai" OR "multimodal model*" OR "vision-language model*" OR "natural language processing" OR "facial recognition" OR "face analysis" OR "phenotypic matching" OR algorithm*)

AND TITLE-ABS-KEY("rare disease*" OR "orphan disease*" OR "rare genetic disease*" OR "rare genetic disorder*" OR "mendelian disease*" OR "mendelian disorder*" OR "undiagnosed disease*")

AND TITLE-ABS-KEY(pediatric* OR paediatric* OR child* OR infant* OR newborn* OR neonat* OR adolescent* OR youth*)

AND TITLE-ABS-KEY(diagnos* OR "differential diagnosis" OR "diagnostic accuracy" OR screening OR "case classification" OR "variant prioritization" OR "variant prioritisation" OR phenotyp* OR "phenotype matching")

AND PUBYEAR > 2014 AND PUBYEAR < 2027

Additional handling: records dated after 1 June 2026 were excluded during export checking if exact-date filtering was not available.

**Web of Science Core Collection (n=201)**

Platform: Web of Science Core Collection, Clarivate. Search date: 26 June 2026. Publication date range: 1 January 2015 to 1 June 2026.

TS=("artificial intelligence" OR "machine learning" OR "deep learning" OR "large language model*" OR LLM OR LLMs OR ChatGPT OR "GPT-4" OR GPT OR "foundation model*" OR "generative AI" OR "multimodal AI" OR "multimodal model*" OR "vision-language model*" OR "natural language processing" OR "facial recognition" OR "face analysis" OR "phenotypic matching" OR algorithm*)

AND TS=("rare disease*" OR "orphan disease*" OR "rare genetic disease*" OR "rare genetic disorder*" OR "Mendelian disease*" OR "Mendelian disorder*" OR "undiagnosed disease*")

AND TS=(pediatric* OR paediatric* OR child* OR infant* OR newborn* OR neonat* OR adolescent* OR youth*)

AND TS=(diagnos* OR "differential diagnosis" OR "diagnostic accuracy" OR screening OR "case classification" OR "variant prioritization" OR "variant prioritisation" OR phenotyp* OR "phenotype matching")

AND PY=(2015-2026)

Additional handling: records dated after 1 June 2026 were excluded during export checking if exact-date filtering was not available.

**CNKI (n=58)**

Platform: China National Knowledge Infrastructure. Search date: 26 June 2026. Publication date range: 1 January 2015 to 1 June 2026.

Search field: Topic (主题). Chinese and English records were eligible if sufficient information was available for screening and data extraction.

(主题 = (人工智能 + 机器学习 + 深度学习 + 大语言模型 + 大型语言模型 + 生成式人工智能 + 多模态 + 多模态模型 + 视觉语言模型 + 自然语言处理 + 面部识别 + 人脸识别 + 表型匹配 + 算法))

AND (主题 = (罕见病 + 少见病 + 孤儿病 + 罕见遗传病 + 罕见遗传性疾病 + 孟德尔病 + 未诊断疾病 + 疑难病))

Source-specific adaptation: A preliminary CNKI search combining artificial intelligence, rare-disease, pediatric, and diagnosis-related concept blocks retrieved very few records. To maximize sensitivity, the final CNKI search used 2 broad topic-based blocks covering artificial intelligence and rare diseases. Pediatric relevance and diagnosis-related use were assessed during title and abstract screening using the same eligibility criteria applied to all other records.

**Wanfang Data (n=444)**

Platform: Wanfang Data Knowledge Service Platform. Search date: 26 June 2026. Publication date range: 1 January 2015 to 1 June 2026.

Search field: Topic (主题). Chinese and English records were eligible if sufficient information was available for screening and data extraction.

(主题:人工智能 OR 机器学习 OR 深度学习 OR 大语言模型 OR 大型语言模型 OR 生成式人工智能 OR 多模态 OR 自然语言处理 OR 人脸识别 OR 面部识别 OR 智能诊断 OR 辅助诊断 )

AND (主题:罕见病 OR 罕见疾病 OR 少见病 OR 孤儿病 OR 罕见遗传病 OR 罕见遗传性疾病 OR 遗传代谢病 OR 单基因病 OR 孟德尔病 OR 未诊断疾病 OR 疑难病)

AND (主题:儿童 OR 儿科 OR 小儿 OR 婴儿 OR 新生儿 OR 青少年)

时间范围: 2015-01-01 至 2026-06-01

AND (主题:诊断 OR 鉴别诊断 OR 辅助诊断 OR 诊断准确性 OR 筛查 OR 病例分类 OR 表型 OR 表型匹配 OR 基因变异优先级排序)

**Additional Sources**

The Cochrane Library was searched as an additional bibliographic database. Hand searching and preprint-server searches were reported separately as other identification methods in the PRISMA-ScR flow diagram. All 4 records identified through these methods were assessed for eligibility and included in the review, contributing 4 of the 81 final included studies.

**Cochrane Library (n=40)**

("rare disease" OR "rare diseases" OR "orphan disease" OR "rare genetic" OR Mendelian OR "undiagnosed disease")

AND (pediatric OR paediatric OR child OR infant OR newborn OR neonate OR adolescent)

AND ("artificial intelligence" OR "machine learning" OR "deep learning" OR "large language model" OR LLM OR ChatGPT OR GPT OR multimodal OR "natural language processing" OR algorithm)

AND (diagnosis OR diagnostic OR screening OR "differential diagnosis" OR "variant prioritization" OR phenotype)

Platform: Cochrane Library, Wiley. Search date: 26 June 2026. Date range: 1 January 2015 to 1 June 2026. Records identified: n=40.

**Preprint Servers (n=2)**

Sources: medRxiv and arXiv. Search date: 26 June 2026. Date range: 1 January 2015 to 1 June 2026.

Search terms combined:

("rare disease" OR "rare diseases" OR "orphan disease" OR "rare genetic" OR Mendelian OR "undiagnosed disease")

AND (pediatric OR paediatric OR child OR infant OR newborn OR neonate OR adolescent)

AND ("artificial intelligence" OR "machine learning" OR "deep learning" OR "large language model" OR LLM OR ChatGPT OR GPT OR multimodal OR "vision-language" OR "natural language processing" OR algorithm)

AND (diagnosis OR diagnostic OR screening OR "differential diagnosis" OR "variant prioritization" OR phenotype)

Records identified: n=2.

Hand Searching (n=2)

Reference lists of included reports and relevant prior reviews were hand searched on 26 June 2026. This process identified 2 records.

Record Management and Study Selection

All records were exported to EndNote for record management. Duplicates were identified using DOI, PMID, title, publication year, and first author; records without DOI or PMID were checked manually by title and bibliographic details. Chinese database records were also checked against English-language records to identify translated or duplicate reports. Of 2557 database records, 494 duplicates were removed, leaving 2063 records for title and abstract screening. A further 4 records were identified through other methods, comprising hand searching (n=2) and preprint servers (medRxiv and arXiv; n=2). These 4 records were assessed separately; none were duplicates of database records, and all 4 were included.

Among the 2063 database records screened, 1957 were excluded at title and abstract screening because they did not evaluate artificial intelligence for diagnosis (n=640), did not address a rare disease or pediatric population (n=615), did not report diagnostic performance (n=520), or were reviews, editorials, conference abstracts, or otherwise outside the review scope (n=182). All 106 reports sought for retrieval were obtained (reports not retrieved, n=0) and assessed in full. Twenty-nine reports were excluded after full-text assessment because they were not focused on rare diseases (n=8), did not report a diagnostic outcome (n=7), did not evaluate an artificial intelligence-based method (n=6), or did not report original empirical research (n=8). This process yielded 77 studies from database searching. Four studies identified through other methods (hand searching, n=2; preprint servers, n=2) were also included, giving a final total of 81 included studies.

**PRISMA-ScR Study Selection Summary**

Identification via databases: records identified, n=2557

Duplicates removed, n=494

Records screened (title/abstract), n=2063

Records excluded at title/abstract screening, n=1957

Not artificial intelligence for diagnosis, n=640

Not rare disease or pediatric population, n=615

No diagnostic performance reported, n=520

Review, editorial, conference abstract, or out of scope, n=182

Reports sought for retrieval, n=106

Reports not retrieved, n=0

Reports assessed for eligibility (full text), n=106

Reports excluded at full-text assessment, n=29

Not focused on rare diseases, n=8

No diagnostic outcome, n=7

Not an artificial intelligence-based study, n=6

Not original empirical research, n=8

Studies included from database searching, n=77

Identification via other methods: records identified, n=4

Hand searching, n=2

Preprint servers (medRxiv and arXiv), n=2

Reports assessed for eligibility from other methods, n=4

Studies included from other methods, n=4

Studies included in review (total), n=81

Targeted Searches of Preprint Servers

Preprint servers were not treated as bibliographic databases for exhaustive retrieval. Instead, we conducted targeted supplementary searches of medRxiv and arXiv on 26 June 2026 to identify recent, highly relevant preprints that might not yet have been indexed in bibliographic databases. These searches were classified as “other methods” in the PRISMA-ScR flow diagram rather than as database searches.

The targeted searches used combinations of the following terms: “rare disease,” “rare diseases,” “Mendelian disease,” “undiagnosed disease,” “pediatric,” “paediatric,” “child,” “children,” “neonate,” “infant,” “adolescent,” “artificial intelligence,” “machine learning,” “deep learning,” “large language model,” “LLM,” and “multimodal AI.” Search results were screened by title and abstract, and potentially eligible records were assessed in full text using the same eligibility criteria as database records. Records already captured by database searches or hand searching were not counted again.

This targeted supplementary search identified 2 additional potentially eligible preprint records: medRxiv (n = 2) and arXiv (n = 0). Both were assessed for eligibility and included in the review. Because this step was intended to identify additional recent records rather than to serve as an exhaustive preprint-server search, raw server-wide hit counts were not incorporated into the database-search totals.
