# Supplementary material for: Machine Learning, Large Language Models, and Multimodal AI for Diagnosing Pediatric Rare Diseases: Scoping Review
Source: J Med Internet Res. 2026 Aug 28;28:e96169. doi: 10.2196/96169 (PMC13524366; doi:10.2196/96169)
Supplement: Supplementary file 2 [file jmir-v28-e96169-s002.docx]

Core characteristics and study-level performance mapping

**Table S1. Core characteristics of the 81 included studies**

| **Ref.** | **Study (year); country** | **Disease or disease group** | **Design and sample** | **AI approach and input** | **Diagnostic task** | **Validation** |
| --- | --- | --- | --- | --- | --- | --- |
| [12] | Şahin et al (2026); Turkey | Inborn errors of immunity | Retrospective cohort; 677 patients | Classical ML: Random Forest, Gradient Boosting, XGBoost, and other ML models; input: lab results | Screening, differential diagnosis | Internal validation, cross-validation |
| [13] | Alsentzer et al (2025); USA | Rare genetic diseases | Simulation/benchmark; N = 465 (UDN) + N = 146 (MyGene2) + N = 1,431 (DDD) | DL: graph neural network (GNN), few-shot learning, SHEPHERD model; input: genomic VCF, phenotype terms | Causal gene discovery, patients-like-me identification, novel disease characterization | Internal validation, external validation |
| [14] | Andriessen et al (2026); Netherlands | ANKRD11-related KBG syndrome | Prospective validation; 93 patients | Facial AI: PhenoScore, Support Vector Machine (SVM), CNN (QMagFace); input: facial photo, phenotypic data | Variant interpretation, classification | Cross-validation, hold-out test |
| [15] | Aref-Eshghi et al (2018); Canada | Neurodevelopmental syndromes | Retrospective cohort; 285 patients | Classical ML: support vector machine (SVM); input: genomic DNA methylation analysis | Concurrent diagnosis, clinical genetic variant classification | Internal validation, cross-validation |
| [16] | Benichou et al (2025); France | Rare genetic syndromes | Retrospective cohort; 4,432 synthetic faces, 1,893 real photographs | Facial AI: Diffusion Models (DPMs), DreamShaper XL Turbo, LoRA, ControlNet, ArcFace R-100; input: facial photo | Classification | Internal validation, external validation |
| [17] | Bolmer et al (2026); Germany | Rare bone diseases | Retrospective cohort; 5,623 hand radiographs | DL: (EfficientNet-B4 backbone); input: hand radiographs | Screening and differential diagnosis | 5-fold cross-validation |
| [18] | Burns et al (2025); USA | Primary ciliary dyskinesia (PCD) | Retrospective cohort; 1,381,809 patients | Classical ML: random forest model; input: insurance claims data | Screening | Fivefold cross-validation |
| [19] | Cao et al (2023); USA | Acute myeloid leukemia (AML) | Retrospective cohort; 2,558 patients | Classical ML: with Support Vector Machine (SVM) and Random Forest (RF) models; input: administrative database | Patient identification and chemotherapy regimen classification | Internal validation with hold-out test sample and external validation with chart-review data source |
| [20] | Chen et al (2024); USA | Pediatric rare disorders | Retrospective cohort; 1,005 patients | Classical ML: Random Forest, XGBoost, Logistic Regression; input: EHR | Classification | Internal validation, external validation |
| [21] | Chen et al (2026); China | All-age rare-disease LLM benchmark; pediatric-specific denominator and performance not separately reported; retained as indirect pediatric-relevant benchmark evidence, not direct pediatric clinical validation. | Simulation/benchmark; 49,760 cases, 22,901 cases | LLM: GPT-4o, Llama3·1-70B, Qwen2·5-72B, etc.; input: clinical text | Rare disease screening (RDS), rare disease confirmation (RDC) | Internal validation, external validation, cross-validation |
| [22] | Chen et al (2022); France | Ciliopathies | Retrospective cohort; 10,447 patients | Classical ML: medical concept embedding, cui_embd_fr, cui2vec, HPO2Vec+; input: electronic health records (EHR) | Screening | Internal validation |
| [23] | Chen et al (2026); China | Retinopathy of prematurity (ROP), infantile retinal diseases | Retrospective cohort; 13,314 patients, 174,540 CFPs | DL: SABPI-Net; input: color fundus photographs (CFPs) | Screening, classification, risk stratification | Internal validation, external validation |
| [24] | Chen et al (2025); China | Chiari malformation type I (CMI), adolescent idiopathic scoliosis (AIS) | Retrospective diagnostic study; 508 patients | DL: ResNet-50 network; input: X-ray images | Screening, differential diagnosis | Internal validation, hold-out test |
| [25] | Cohen et al (2022); USA | Pediatric rare diseases | Retrospective cohort; 1,083 patients | Classical ML: Exomiser and AMELIE; input: genomic VCF | Differential diagnosis | Internal validation, expert review |
| [26] | Crisafulli et al (2024); Italy | X-linked hypophosphatemia (XLH) | Retrospective cohort; 431,021 patients | Classical ML: (Random Forest, Recursive Partitioning and Regression Tree (RPART)); input: EHR | Differential diagnosis | Internal validation |
| [27] | Cuce et al (2026); Turkey | Idiopathic intracranial hypertension | Retrospective cohort; 124 patients | Classical ML: supervised ML with random forest, support vector machine, multilayer perceptron, XGBoost, k-nearest neighbors, and Bagging; input: MRI | Classification | Internal validation with 20 repeated nested cross-validation splits |
| [28] | Spencer et al (2026); Democratic Republic of the Congo | Sickle Cell Disease | Retrospective cohort; 308 patients, 154 SCD, 154 controls | Facial AI: ML, support vector machine; input: facial photo | Screening, differential diagnosis | Leave-one-out cross-validation |
| [29] | De Furia et al (2025); Canada | Congenital hypothyroidism | Retrospective cohort; 616,910 cases | Classical ML: with RUSBoost and Gaussian Noise resampling; input: lab metabolomics (TSH levels) | Screening | Stratified 5-fold cross-validation |
| [30] | De La Vega et al (2021); USA | Rare genetic diseases | Retrospective cohort; 119 cases | Classical ML: Bayesian, AI-based gene prioritization tool (GEM); input: genomic VCF | Differential diagnosis | Internal validation |
| [31] | del Rincón et al (2025); Spain | PACS1 syndrome, DEE66, NOCGUS, BRWS1 | Retrospective cohort; 38 patients | Facial AI: DeepGestalt, GestaltMatcher, Deep CNN (DCNN); input: facial photo | Differential diagnosis, classification | Internal validation, physician comparison |
| [32] | Dingemans et al (2023); Netherlands | Rare genetic diseases | Retrospective cohort; 711 patients | Facial AI: ML with support vector machine (SVM) and deep neural network (VGGFace2); input: facial photo and clinical features | Classification and differential diagnosis | Nested cross-validation and external validation |
| [33] | Echeverry-Quiceno et al (2023); Colombia | Down syndrome, Morquio syndrome, Noonan syndrome, Neurofibromatosis type 1 | Cross-sectional; 130 participants (79 controls, 51 patients) | Facial AI: Face2Gene DL algorithm; Euclidean Distance Matrix Analysis for facial phenotyping; input: facial photo (2D images, 18 landmarks) | Differential diagnosis, facial phenotyping | Physician comparison, population-specific validation in admixed Colombian cohort |
| [34] | Elmas and Gogus (2020); Turkey | Rare genetic diseases | Retrospective cohort; 25 patients | Facial AI: DeepGestalt, Face2Gene; input: facial photo, clinical text | Differential diagnosis, classification | Physician comparison |
| [35] | Fadul et al (2026); United Arab Emirates | Mucopolysaccharidoses (MPS) | Retrospective cohort; 106 patients | DL: Graph Neural Networks (GNNs), Graph Convolutional Networks (GCNs), Graph Attention Networks (GATs), GraphSAGE, Chebyshev Convolutional Networks (CCNs); input: Electronic Health Records (EHR) | Classification | Nested cross-validation |
| [36] | Feng et al (2025); China | Infantile Epileptic Spasms Syndrome (IESS) | Retrospective cohort; 129 patients | Multimodal AI: DL, IESS-FusionNet; input: EEG and EMG | Detection | Internal validation |
| [37] | Garcelon et al (2018); France | Rett syndrome, Lowe syndrome, Silver Russell syndrome, Bardet-Biedl syndrome, DOCK8 deficiency, Activated PI3-kinase Delta Syndrome (APDS) | Retrospective cohort; 446,481 patients | Classical ML: with UMLS and TF-IDF; input: clinical text | Phenotype identification and classification | Internal validation with expert evaluation |
| [38] | Greco et al (2026); USA | Pulmonary hypertension, severe asthma | Retrospective cohort; 14,305 patients | DL: weakly supervised transformer (WEST) framework; input: EHR | Disease diagnosis, subphenotyping | Cross-validation |
| [39] | Groen et al (2025); Netherlands | Glycogen storage disease type Ia | Retrospective cohort; 3,958 patients | Classical ML: gradient-boosted tree algorithms (CatBoost, XGBoost, Random Forest); input: lab metabolomics (acylcarnitine profiles) | Screening and classification | Nested cross-validation |
| [40] | Hack et al (2024); USA | SCN8A-related epilepsy | Retrospective cohort; 180 patients | Classical ML: random forest, penalized ordinal logistic regression, stacked meta-learner; input: genomic VCF | Classification, risk stratification | Internal validation, external validation |
| [41] | Hennocq et al (2023); France | Treacher Collins syndrome, other rare craniofacial malformations | Prospective validation; NR | Facial AI: Faster R-CNN, patch-based active appearance model (AAM), constrained local model (CLM); input: facial photographs | Classification, phenotyping | Internal validation, cross-validation |
| [42] | Herr et al (2024); USA | Rare genetic diseases | Retrospective cohort; 659,139 patients | Classical ML: rule-based NLP (NOBLE Coder) and ML-based NLP (ScispaCy); input: electronic health records (EHRs) | Screening and classification of rare genetic diseases | Internal validation and manual chart review |
| [43] | Hinterwimmer et al (2022); Germany | Ewing sarcoma, acute osteomyelitis | Cross-sectional; 42,608 radiographs | DL: ResNet50, self-supervised learning; input: radiographs | Differential diagnosis | Cross-validation |
| [44] | Hong et al (2021); China | Genetic syndromes | Cross-sectional; 456 frontal facial photos | Facial AI: DL, VGG-16 architecture; input: facial photo | Screening | Five-fold cross-validation, physician comparison |
| [45] | Hsieh et al (2022); Germany | Rare genetic disorders | Prospective validation; 17,560 patients, 26,065 images | Facial AI: deep CNN (DCNN), encoder-classifier composition; input: facial photo | Differential diagnosis, classification | Internal validation, cross-validation |
| [46] | Huang et al (2024); China | Williams-Beuren syndrome | Retrospective cohort; 954 images | Facial AI: deep CNNs (CNNs), EfficientNet-b3, ResNet-50, VGG-16, VGG-16BN, VGG-19, VGG-19BN; input: facial photo | Differential diagnosis | Five-fold cross-validation, external validation |
| [47] | Huang et al (2023); USA | Turner syndrome | Retrospective cohort and prospective validation; 466 patients | Classical ML: computable phenotype algorithm; input: electronic health records (EHR) | Identification of Turner syndrome cases | Internal validation and external validation |
| [48] | Iadanza et al (2020); Italy | Inherited retinal diseases, retinitis pigmentosa | Prospective validation; 38 patients | Classical ML: support vector machines (SVMs); input: pupillometry | Classification, screening | Leave-one-out cross-validation |
| [49] | Ilić et al (2025); Serbia | Skeletal dysplasias | Prospective diagnostic accuracy study; 45 patients | LLM: ChatGPT (GPT-4) and DeepSeek Medical AI; input: structured clinical vignettes | Differential diagnosis | Physician comparison |
| [50] | Jaroy et al (2024); Norway | Hirschsprung's disease | Retrospective cohort; 178 patients | Classical ML: random forest, gradient-boosted classifier; input: EHR | Risk stratification | 5-fold stratified cross-validation |
| [51] | Song et al (2025); China | Rare genetic diseases | Retrospective cohort; 500 questions | LLM: Graph RAG, Cypher RAG, Vector RAG; input: text | Differential diagnosis | Internal validation |
| [52] | Jiang et al (2025); China | Biliary atresia | Retrospective cohort and prospective validation; 348 patients | DL: logistic regression, Lasso, and DL; input: ultrasound images and serum MMP-7 values | Screening and differential diagnosis | Internal validation and external validation |
| [53] | Kadi et al (2024); France | PAX9, WNT10A, EDA, and other rare genetic dental diseases | Prospective validation; 272 dental panoramic X-rays | DL: U-Net, Mask R-CNN, DL; input: dental panoramic X-rays | Differential diagnosis, classification | Internal validation, cross-validation |
| [54] | Kauffman et al (2025); USA | Neonatal culture-negative sepsis, postoperative acute kidney injury | Retrospective cohort; 8,015 patients | DL: Deep Geometric Learning, Graph Neural Network (GNN); input: Electronic Health Records (EHR) | Risk stratification, differential diagnosis | Internal validation, external validation |
| [55] | Kothari et al (2022); USA | PTEN hamartoma tumor syndrome (PHTS) | Retrospective cohort; 1,215 patients | Classical ML: computational phenotype, ML algorithms; input: EHR | Screening for genetic testing | Internal validation, physician comparison |
| [56] | Li et al (2026); China | Biliary atresia | Retrospective diagnostic study; 50 patients | Classical ML: logistic regression and random forest classifiers; input: clinical and laboratory data | Differential diagnosis | Internal validation with repeated stratified 5-fold cross-validation |
| [57] | Li et al (2026); China | Eosinophilic esophagitis | Cross-sectional; NR | Classical ML: including Scissor, LASSO, and random forest; input: single-cell RNA sequencing | Molecular diagnostics | Internal validation |
| [58] | Li et al (2025); China | Inborn errors of metabolism (IEMs) | Retrospective cohort; 355,197 cases | Classical ML: Random Forest, SHAP (SHapley Additive exPlanations); input: lab metabolomics (GC-MS urinary organic acid profiles) | Screening, differential diagnosis | Internal validation, 100 repetitions with 95% confidence interval |
| [59] | Ma et al (2025); China | Biliary atresia | Retrospective cohort and prospective validation; 681 BA patients, 898 non-BA patients | Multimodal AI: DL, Swin Transformer V2, self-masked attention mechanism; input: ultrasound images, clinical data, laboratory results | Screening and classification | Internal validation, external validation |
| [60] | Mao et al (2025); China | Rare diseases | Retrospective cohort; 2,271 cases | Classical ML: BERT-based NLP model, PhenoBrain AI pipeline; input: Electronic Health Records (EHRs) | Differential diagnosis | Internal validation, external validation, physician comparison |
| [61] | Méndez Barrera et al (2023); Mexico/USA | Primary immune deficiencies | Proof-of-concept data mining study; 2,396 patients | Classical ML: Extreme Gradient Boosting (XGBoost) with SHAP for feature importance; input: clinical and laboratory features | Differential diagnosis prediction | Internal validation with random train/test split |
| [62] | Mohammed et al (2026); China | Biliary atresia | Prospective validation; 1,439 patients, 4,546 sonographic gallbladder images | DL: self-supervised learning, Swapping Assignments between Views (SwAV) method, lightweight CNNs; input: sonographic images | Classification | Internal validation, external validation |
| [63] | Orenbuch et al (2025); USA | Severe developmental disorders | Retrospective cohort; 31,058 cases | Classical ML: deep generative model, popEVE; input: genomic VCF | Variant effect prediction, disease diagnosis | Internal validation, external validation |
| [64] | Porfireva et al (2025); Russia | Primary immunodeficiencies | Retrospective cohort; 4,825 missense variants | Classical ML: MultiPASS software; input: genomic VCF | Pathogenicity prediction | Leave-one-out cross-validation, 5-fold cross-validation |
| [65] | Porras et al (2021); USA | Genetic syndromes | Retrospective cohort; 2,800 facial photographs | Facial AI: deep neural networks, facial statistical shape models; input: facial photo | Screening, risk stratification | Cross-validation |
| [66] | Rassmann et al (2023); Germany | Skeletal dysplasias (achondroplasia, hypochondroplasia, pseudohypoparathyroidism, Noonan, Silver-Russell, Ullrich-Turner syndromes) | Retrospective validation; 568 radiographs from 189 patients (GDBD); RSNA dataset 12,611 training and 200 test images | DL: CNN ensemble (EfficientNet-based); input: hand and wrist X-ray | Bone age assessment | Internal validation on RSNA test set, external validation on GDBD and DHA cohorts |
| [67] | Reiley et al (2023); USA | Mendelian disorders | Retrospective cohort; 25 trios | Classical ML: NLP, Microsoft Text Analytics for Health, Exomiser; input: genomic VCF, phenotypic data from medical records | Differential diagnosis | Internal validation |
| [68] | Rider et al (2021); United States | Primary immunodeficiency diseases | Retrospective cohort; 1,762 patients | Classical ML: Bayesian network (BN) model; input: electronic health record (EHR) | Risk stratification and differential diagnosis | Internal validation and external validation |
| [69] | Robinson et al (2020); United Kingdom | Juvenile-onset systemic lupus erythematosus | Retrospective cohort; 67 patients, 39 healthy controls | Classical ML: balanced random forest (BRF) and sparse partial least squares-discriminant analysis (sPLS-DA); input: immunophenotyping profiles from flow cytometry | Classification, risk stratification | Ten-fold cross-validation |
| [70] | Rudy et al (2025); USA | Rare genetic disorders | Retrospective cohort; 19 patients | Facial AI: DeepGestalt and Feature Matcher; input: facial photo | Differential diagnosis | Physician comparison |
| [71] | Salvia et al (2025); India | Rare diseases, including Gaucher, ALS, and AADC deficiency | Retrospective cohort; 170,000+ patients | DL: Hierarchical Temporal Transformer (HTT) model; input: electronic health records (EHR) | Early detection of rare diseases | Internal validation, external validation |
| [72] | Schmidt et al (2024); Germany | Ultrarare genetic disorders | Prospective cohort; 1,577 patients | Facial AI: YieldPred (ML for exome diagnostic yield prediction), GestaltMatcher/PEDIA (facial image analysis); input: genomic VCF (exome sequencing), facial images | Differential diagnosis, variant prioritization, diagnostic yield prediction | Prospective national cohort, internal validation with held-out test set |
| [73] | Schwitzkowski et al (2026); Austria/Germany | Inborn errors of immunity/primary immunodeficiency (IEI/PID) | Retrospective cohort; 825 patients | Classical ML: with random forest (RF) algorithm; input: structured patient data | Classification, differential diagnosis | Internal validation, cross-validation |
| [74] | Song et al (2025); China | Rare genetic diseases | Retrospective cohort; 509 publications, 1,147 data records | Facial AI: TabNet, Node, TabTransformer, FTTransformer; input: tabular dataset, text | Diagnostic models for rare genetic diseases | Internal validation, cross-validation |
| [75] | Ustach et al (2026); USA | Mendelian disorders | Retrospective cohort; 9,989 cases | Classical ML: random forest (RF) model with ensemble of multiple similarity scores; input: genomic VCF | Differential diagnosis | Internal validation |
| [76] | van der Donk et al (2019); Netherlands | Rare genomic neurodevelopmental disorders, intellectual disability | Retrospective cohort and prospective validation; 71 patients with Koolen-de Vries syndrome, 14 patients with PACS1, 11 patients with PPM1D, 16 patients with PHIP, and 71-80 matched ID controls | Facial AI: hybrid model combining Clinical Face Phenotype Space (CFPS) and OpenFace; input: facial photo | Detection of facial gestalt and classification of rare ID syndromes | Internal validation and cross-validation |
| [77] | Vargova et al (2025); Spain | Hirschsprung disease | Retrospective cohort; 218 patients | DL: DenseNet121; input: contrast enema images | Classification | Internal validation |
| [78] | Wang et al (2026); China | Rare diseases | Retrospective cohort; NR patients | LLM: driven multi-agent system; input: clinical text | Screening and differential diagnosis | Internal validation |
| [79] | Wang and Lü (2020); China | Rare genetic diseases | Simulation/Benchmark; 1,100 patients | Classical ML: Semantic similarity algorithm using opposite phenotypes; input: Clinical text | Auxiliary diagnosis | Internal validation |
| [80] | Xie et al (2025); USA | Inherited metabolic disorders (IMDs) | Retrospective cohort; 119 patients | Multimodal AI: ML with Random Forest classifier; input: genomic VCF, metabolomics | Screening and differential diagnosis | Internal validation |
| [81] | Yahya et al (2025); Bulgaria | Genetic disorders, dysmorphic syndromes | Retrospective cohort; 151 patients | Facial AI: DL, Face2Gene; input: facial photo | Differential diagnosis, classification | Internal validation |
| [82] | Yang et al (2021); China | Inherited Metabolic Diseases | Retrospective Cohort; 5,907,547 | Classical ML: Random Forest, Gradient Boosting; input: Lab metabolomics | Screening, Risk Stratification | Internal Validation, Physician Comparison |
| [83] | Yang et al (2024); China | Inherited metabolic diseases | Retrospective cohort; 309,102 patients | Classical ML: with 9 algorithms; input: lab metabolomics | Screening | Internal validation |
| [84] | Yang et al (2022); United States | Noonan syndrome | Retrospective cohort; 189 patients, 162 NS cases, 16,200 controls | DL: CNN (CNN); input: electronic health records (EHR) | Screening | Internal validation, external validation |
| [85] | Yates et al (2021); USA | Rare diseases | Observational study; 1,042 patients | Classical ML: Louvain community detection, support vector classifier; input: phenotypic data annotated by Human Phenotype Ontology terms | Cluster analysis, classification | Internal validation, 10-fold cross-validation |
| [86] | Young et al (2024); USA | Rare pediatric genetic diseases | Retrospective cohort; 61 cases | LLM: GPT-4, GPT-4 HPO; input: clinical text | Differential diagnosis | Fisher's exact test |
| [87] | Yu et al (2024); China | DiGeorge syndrome, Down syndrome, Williams syndrome | Cross-sectional; 109 patients | Facial AI: ML, Dlib, ResNet; input: facial photo | Classification | 10-fold cross-validation |
| [88] | Zaunseder et al (2024); Germany | Glutaric aciduria type 1 | Retrospective cohort; 1,025,953 newborns | Classical ML: logistic regression, ridge regression, support vector machine; input: lab metabolomics | Screening | Internal validation, cross-validation |
| [89] | Zhang et al (2016); China | Duchenne muscular dystrophy | Retrospective diagnostic study; 30 participants; 485 MRI images | Classical ML: Wavelet feature extraction, principal component analysis, and support vector machine with radial basis function kernel; input: MRI (T1-weighted and T2-weighted images) | MRI-based classification and differential diagnosis of DMD | Internal validation using 10-fold cross-validation |
| [90] | Zhao et al (2026); China | Rare diseases (2,919 diseases spanning 14 medical specialties) | Retrospective benchmark evaluation; 6,401 clinical cases covering 2,919 rare diseases | LLM: DeepRare, multi-agent LLM-based system integrating >40 specialized tools; input: clinical text, HPO terms, genomic VCF | Differential diagnosis | Internal validation, external validation across nine datasets |
| [91] | Zheng et al (2026); China | Growth hormone deficiency, idiopathic central precocious puberty | Retrospective cohort; 274 patients | Multimodal AI: DL, ADHTransNet; input: MRI | Hormone prediction | Internal validation, cross-validation |
| [92] | Zucca et al (2024); Italy | Genetic diseases | Simulation/benchmark; 35 patients | Classical ML: eVai pathogenicity score, phenotypic similarity score; input: genomic VCF | Variant prioritization, classification | Internal validation, Leave-One-Proband-Out Cross Validation |

Abbreviations: AI, artificial intelligence; DL, deep learning; EHR, electronic health record; LLM, large language model; ML, machine learning; MRI, magnetic resonance imaging; NR, not reported.

Note: Country reflects the reported or inferred research setting. Validation descriptions are reproduced in condensed form from the source reports. Study labels use both author surnames for references with two authors and the first author followed by et al for references with three or more authors; the publication year is shown for every entry.

**Table S2. Study-level mapping of diagnostic tasks, evaluation samples, and reported performance**

Each row represents one included study. Total sample sizes and performance results are reproduced in condensed form from the source reports. A separate evaluation denominator is reported only when it was explicitly identifiable from the extracted information; otherwise it is coded as NR. No denominators or performance values were imputed.

| **Ref.** | **Study (year) and diagnostic task** | **Disease or disease group** | **Sample size and Reported evaluation set or denominator** | **Reference standard/comparator and validation** | **Reported metric(s)** | **Reported result(s)** |
| --- | --- | --- | --- | --- | --- | --- |
| [12] | Şahin et al (2026) | inborn errors of immunity | Total: 677 patients Evaluation denominator: NR | Reference/comparator: IEI diagnosis according to IUIS classification criteria Validation: internal validation, cross-validation | AUROC/AUC; Sensitivity/recall; Specificity | The Random Forest model achieved an AUROC of 0.86, sensitivity of 0.81, and specificity of 0.75. Key predictors included lymphocyte count, platelet count, and neutrophil count. |
| [13] | Alsentzer et al (2025) | rare genetic diseases | Total: N = 465 (UDN) + N = 146 (MyGene2) + N = 1,431 (DDD) Evaluation denominator: NR | Reference/comparator: gold standard diagnosis Validation: internal validation, external validation | Sensitivity/recall | SHEPHERD achieved average recall at k of 0.69 when k = 3 and 0.85 when k = 5 on the EXPERT-CURATED gene lists, and 0.21, 0.38, and 0.48 for k = 1, 5, and 10, respectively, on the VARIANT-FILTERED gene lists. SHEPHERD outperformed baseline approaches in causal gene discovery and patients-like-me identification. |
| [14] | Andriessen et al (2026) | ANKRD11-related KBG syndrome | Total: 93 patients Evaluation denominator: NR | Reference/comparator: clinical geneticist's judgment, functional studies Validation: cross-validation, hold-out test | AUROC/AUC; Accuracy; Brier score | PhenoScore achieved high predictive accuracy with an AUC of 0.95 and a Brier score of 0.089; mean PhenoScore prediction score for clinically confirmed pathogenic missense variants was 0.94. |
| [15] | Aref-Eshghi et al (2018) | neurodevelopmental syndromes | Total: 285 patients Evaluation denominator: NR | Reference/comparator: American College of Medical Genetics Guidelines Validation: internal validation, cross-validation | Accuracy; Sensitivity/recall; Specificity | 99.6% accuracy, 100% sensitivity and specificity in predicting disease class |
| [16] | Benichou et al (2025) | rare genetic syndromes | Total: 4,432 synthetic faces, 1,893 real photographs Evaluation denominator: NR | Reference/comparator: expert dysmorphologists' diagnosis Validation: internal validation, external validation | AUROC/AUC; Accuracy; Top-k performance | Top-1 accuracy: 0.823, 0.869, 0.971; AUC: 0.991, 0.993; improved performance in ultra-rare syndromes and Asian individuals |
| [17] | Bolmer et al (2026) | rare bone diseases | Total: 5,623 hand radiographs Evaluation denominator: NR | Reference/comparator: gold standard diagnosis Validation: 5-fold cross-validation | Balanced accuracy; Sensitivity/recall; Specificity; F-score; Top-k performance | The affected vs. unaffected classifier achieved an overall accuracy of 85.5%, a mean F1-score of 84.6%, a sensitivity of 82.7%, and a specificity of 90.8% on the test set. The ten-class disorder classifier reached a balanced accuracy of 76.6% and Top-3 accuracies above 90% across ten clinically relevant groups. |
| [18] | Burns et al (2025) | primary ciliary dyskinesia (PCD) | Total: 1,381,809 patients Evaluation denominator: NR | Reference/comparator: PCD Foundation Registry (PCDFR) Validation: fivefold cross-validation | Sensitivity/recall; PPV/precision | Positive predictive value: 0.45-0.54, Sensitivity: 0.75-0.94, 7705 patients classified as positive |
| [19] | Cao et al (2023) | acute myeloid leukemia (AML) | Total: 2,558 patients Evaluation denominator: NR | Reference/comparator: manual review of pharmacy data Validation: internal validation with hold-out test sample and external validation with chart-review data source | Sensitivity/recall; PPV/precision | The selected SVM model yielded a sensitivity of 0.97 and a positive predictive value (PPV) of 0.97 in the hold-out test sample. The selected RF model yielded overall PPV greater than or equal to 0.88 and sensitivity greater than or equal to 0.86 across all courses in the test sample. |
| [20] | Chen et al (2024) | pediatric rare disorders | Total: 1,005 patients Evaluation denominator: NR | Reference/comparator: genetic specialists' decisions Validation: internal validation, external validation | AUROC/AUC; AUPRC/PR-AUC | AUROC 0.823, AUPRC 0.918, AUROC 0.947, AUPRC 0.972 |
| [21] | Chen et al (2026) | rare diseases, 4597 disorder concepts, 3522 disorder concepts | Total: 49,760 cases, 22,901 cases Evaluation denominator: NR | Reference/comparator: Orphanet, a well-acknowledged rare disease knowledge graph Validation: internal validation, external validation, cross-validation | Sensitivity/recall; Top-k performance | GPT-4o achieved a top-1 recall of 33.1% and a top-5 recall of 56.9% in RDS, and a top-1 recall of 64.2% and a top-5 recall of 85.9% in RDC. |
| [22] | Chen et al (2022) | ciliopathies | Total: 10,447 patients Evaluation denominator: NR | Reference/comparator: diagnosed ciliopathy cases Validation: internal validation | Rank; Enrichment | The proposed approach achieved a 12.2-fold improvement in the enrichment of ciliopathy patients among the top-ranked patients compared to random testing. The minimum number of patients that would need to be screened to detect 30 out of 253 ciliopathy patients was 102 with the proposed method, compared to 1244 with random testing. |
| [23] | Chen et al (2026) | retinopathy of prematurity (ROP), infantile retinal diseases | Total: 13,314 patients, 174,540 CFPs Evaluation denominator: NR | Reference/comparator: International Classification of Retinopathy of Prematurity Validation: internal validation, external validation | Accuracy | SABPI-Net achieved superior performance compared to 13 SOTA methods, with 95.32% accuracy on mainstream clinical tasks, 73.58% on ROP five-stage classification, and 84.25% on multi-disease classification. |
| [24] | Chen et al (2025) | Chiari malformation type I (CMI), adolescent idiopathic scoliosis (AIS) | Total: 508 patients Evaluation denominator: NR | Reference/comparator: spine surgery experts, MRI Validation: internal validation, hold-out test | Accuracy; Sensitivity/recall; Specificity | The ResNet-50 Coronal model achieved 94.00% accuracy, 90.63% sensitivity, and 100.00% specificity in scoliosis diagnosis. The ResNet-50 Dual model achieved 84.00% accuracy, 58.33% sensitivity, and 92.11% specificity in CMS diagnosis. |
| [25] | Cohen et al (2022) | pediatric rare diseases | Total: 1,083 patients Evaluation denominator: NR | Reference/comparator: clinical genetic testing, expert review Validation: internal validation, expert review | Diagnostic rate | Diagnostic rates: 11% in patients with prior negative genetic testing, 34.5% in naive patients. Incorporating SVs from genome sequencing added up to 13% of new diagnoses in previously unsolved cases. HiFi-GS yielded increased discovery rate with >4-fold more rare coding SVs compared with srGS. |
| [26] | Crisafulli et al (2024) | X-linked hypophosphatemia (XLH) | Total: 431,021 patients Evaluation denominator: NR | Reference/comparator: clinical diagnosis recorded by family pediatricians Validation: internal validation | AUROC/AUC; Accuracy | The three models achieved a fair diagnostic accuracy, with Random Forest yielding the highest AUC values in both one-year and two-years prior to ID analyses. The selected predictors of XLH diagnosis were factors associated with the number of vitamin D prescriptions, acute respiratory infections, antihistamines for systemic use prescriptions, and allergology visit prescriptions. |
| [27] | Cuce et al (2026) | idiopathic intracranial hypertension | Total: 124 patients Evaluation denominator: NR | Reference/comparator: lumbar puncture and neuroophthalmologic assessment Validation: internal validation with 20 repeated nested cross-validation splits | AUROC/AUC; AUPRC/PR-AUC; Sensitivity/recall; PPV/precision | mean AUC values clustered around 0.80-0.82 for Bagging, KNN, MLP, random forest, and XGBoost, with similar F1-scores and precision-recall profiles and no statistically significant pairwise differences after multiple-comparison correction. |
| [28] | Spencer et al (2026) | Sickle Cell Disease | Total: 308 patients, 154 SCD, 154 controls Evaluation denominator: NR | Reference/comparator: gel electrophoresis Validation: leave-one-out cross-validation | AUROC/AUC; Accuracy; Sensitivity/recall; Specificity | 79.5% accuracy, 84.3% AUROC, 81.2% sensitivity, 77.9% specificity |
| [29] | De Furia et al (2025) | congenital hypothyroidism | Total: 616,910 cases Evaluation denominator: NR | Reference/comparator: current TSH-based screening process Validation: stratified 5-fold cross-validation | Sensitivity/recall; PPV/precision | The best-performing model achieved a PPV of 16.8% and a sensitivity of 100% across 5 folds of stratified cross-validation, representing a 60% improvement over the current first-tier screening process. |
| [30] | De La Vega et al (2021) | rare genetic diseases | Total: 119 cases Evaluation denominator: NR | Reference/comparator: manual filtering and variant prioritization approaches Validation: internal validation | Sensitivity/recall; Top-k performance; Rank | GEM ranked 97% of previously reported causal gene(s) and variant(s) among the top 10 candidates, with 92% in the top 2. GEM's recall was essentially unaltered in the absence of parental genotypes. |
| [31] | del Rincón et al (2025) | PACS1 syndrome, DEE66, NOCGUS, BRWS1 | Total: 38 patients Evaluation denominator: NR | Reference/comparator: clinical evaluation, Human Phenotype Ontology (HPO) Validation: internal validation, physician comparison | AUROC/AUC | AUC values ranged from 0.406 to 0.996, indicating potential facial overlap among PACS1-, PACS2-, and WDR37-related syndromes. |
| [32] | Dingemans et al (2023) | rare genetic diseases | Total: 711 patients Evaluation denominator: NR | Reference/comparator: OMIM-listed disorders and expert opinion Validation: nested cross-validation and external validation | AUROC/AUC; Brier score | PhenoScore achieved a median AUC of 0.91 and Brier score of 0.13 for classifying genetic syndromes, outperforming existing methods such as Phenomizer and LIRICAL. |
| [33] | Echeverry-Quiceno et al (2023) | Down syndrome, Morquio syndrome, Noonan syndrome, Neurofibromatosis type 1 | Total: 130 participants (79 controls, 51 patients) Evaluation denominator: NR | Reference/comparator: clinical diagnosis based on genetic testing Validation: physician comparison, population-specific validation in admixed Colombian cohort | Accuracy | Face2Gene diagnostic accuracy: Down syndrome 100%, Noonan syndrome 66.7% (vs 100% in European population), Morquio and NF1 below 10%; 58.2% and 65.4% of facial traits significantly different in DS and MS respectively. |
| [34] | Elmas and Gogus (2020) | rare genetic diseases | Total: 25 patients Evaluation denominator: NR | Reference/comparator: whole-exome sequencing (WES) results Validation: physician comparison | Case match rate | The Face2Gene application listed possible preliminary diagnoses (30 disease suggestions). Of these, 12 (48%) cases were correctly matched. The most common disease group in the patients was neurological disease (96%). |
| [35] | Fadul et al (2026) | Mucopolysaccharidoses (MPS) | Total: 106 patients Evaluation denominator: NR | Reference/comparator: gold standard diagnosis Validation: nested cross-validation | AUROC/AUC; F-score | The GCN model with feature set selected by chi-square FS method reported a superior performance in effectively distinguishing between control and MPS patients with an AUC of 0.97 and F1 score of 0.93 [95% CI (0.91, 1.00)]. |
| [36] | Feng et al (2025) | Infantile Epileptic Spasms Syndrome (IESS) | Total: 129 patients Evaluation denominator: NR | Reference/comparator: expert visual review of EEG and EMG Validation: internal validation | Accuracy; Sensitivity/recall; Specificity | 89.5% accuracy, 90.7% specificity, 88.3% sensitivity |
| [37] | Garcelon et al (2018) | Rett syndrome, Lowe syndrome, Silver Russell syndrome, Bardet-Biedl syndrome, DOCK8 deficiency, Activated PI3-kinase Delta Syndrome (APDS) | Total: 446,481 patients Evaluation denominator: NR | Reference/comparator: expert evaluation and Orphadata Validation: internal validation with expert evaluation | PPV/precision | Average precision: 0.79-0.91; Mean average precision: 0.79; 72% of phenotypes were not negated; 4% of phenotypes were related to family history |
| [38] | Greco et al (2026) | pulmonary hypertension, severe asthma | Total: 14,305 patients Evaluation denominator: NR | Reference/comparator: gold-standard labels from expert physician chart review Validation: cross-validation | AUROC/AUC; Specificity; PPV/precision; F-score | WEST achieved the highest AUC, F1 score, PPV, and specificity across all methods, with improved performance with the number of gold-standard training labels. |
| [39] | Groen et al (2025) | glycogen storage disease type Ia | Total: 3,958 patients Evaluation denominator: NR | Reference/comparator: gold standard diagnosis Validation: nested cross-validation | AUROC/AUC; AUPRC/PR-AUC; F-score; False-positive outcome | The model achieved a mean ROC AUC of 0.955, PR AUC of 0.674, and F4-score of 0.488 in nested CV. On the held-out test set, the model correctly identified 5 out of 6 GSD Ia patients with only 4 false positives. |
| [40] | Hack et al (2024) | SCN8A-related epilepsy | Total: 180 patients Evaluation denominator: NR | Reference/comparator: electrophysiological score, clinical severity Validation: internal validation, external validation | Accuracy; MAE/MAD/RMSE | Unsupervised Stacked model: mean absolute error 0.06, root-mean-square error 0.16, percentage of correct classification 94.8%; Supervised p-ORM: mean absolute error 0.144, root-mean-square error 0.54, percentage of correct classification 81.0% |
| [41] | Hennocq et al (2023) | Treacher Collins syndrome, other rare craniofacial malformations | Total: NR Evaluation denominator: NR | Reference/comparator: manual landmarking, ground-truth data Validation: internal validation, cross-validation | Annotation error | The best model was the patch-based AAM with a final error of 0.033 (+/-0.021) for frontal faces, 0.053 (+/-0.076) for profile faces, and 0.450 (+/-0.045) for ears. The pretrained models based on public photographs did not improve the annotation performance. |
| [42] | Herr et al (2024) | rare genetic diseases | Total: 659,139 patients Evaluation denominator: NR | Reference/comparator: manual chart review and Orphanet Validation: internal validation and manual chart review | Sensitivity/recall; PPV/precision; Prevalence | The rule-based NLP method achieved a precision of 97.5% and a recall of 90.3% in identifying rare genetic disease diagnoses. The machine learning-based NLP method had a precision of 73.5% and a recall of 82.0%. The study identified 37,326 patients with rare genetic disease diagnoses, representing a prevalence of 5.66% in the cohort. |
| [43] | Hinterwimmer et al (2022) | Ewing sarcoma, acute osteomyelitis | Total: 42,608 radiographs Evaluation denominator: NR | Reference/comparator: gold standard diagnosis Validation: cross-validation | Accuracy | Accuracy: 81.5%/54.2% (untrained), 89.6%/70.8% (ImageNet-pretrained), 94.0%/88.1% (transfer learning approach) |
| [44] | Hong et al (2021) | genetic syndromes | Total: 456 frontal facial photos Evaluation denominator: NR | Reference/comparator: gold standard diagnosis by karyotyping, array comparative genomic hybridization, or next-generation sequencing Validation: five-fold cross-validation, physician comparison | AUROC/AUC; Accuracy; Sensitivity/recall; Specificity; F-score | The VGG-16 model achieved an accuracy of 0.8860 +/- 0.0211, specificity of 0.9124 +/- 0.0308, recall of 0.8597 +/- 0.0190, F1-score of 0.8829 +/- 0.0215, and an area under the receiver operating characteristic curve of 0.9443 +/- 0.0276. |
| [45] | Hsieh et al (2022) | rare genetic disorders | Total: 17,560 patients, 26,065 images Evaluation denominator: NR | Reference/comparator: clinically or molecularly confirmed diagnoses Validation: internal validation, cross-validation | Accuracy; Top-k performance; Rank | GestaltMatcher achieved 64.30% and 86.59% accuracy within the top-10 and top-30 ranks, respectively, when using the frequent gallery, and 48 of 91 subjects were matched, and 40 of 79 families were connected when using the top-30 rank. |
| [46] | Huang et al (2024) | Williams-Beuren syndrome | Total: 954 images Evaluation denominator: NR | Reference/comparator: expert diagnosis Validation: five-fold cross-validation, external validation | Accuracy; Sensitivity/recall; Specificity; PPV/precision; F-score | The VGG-19BN model achieved the best performance with an accuracy of 95.10%, precision of 100%, recall of 83.87%, specificity of 93.42%, and F1 score of 91.23%. The model outperformed human experts in all metrics. |
| [47] | Huang et al (2023) | Turner syndrome | Total: 466 patients Evaluation denominator: NR | Reference/comparator: chart review Validation: internal validation and external validation | Accuracy; Sensitivity/recall; Specificity; PPV/precision; NPV | The computable phenotype algorithm had an average C-statistic of 0.93 across all sites, with sensitivity of 97.0%, specificity of 87.9%, PPV of 93.6%, NPV of 90.0%, accuracy of 93.6%, and C-statistic of 0.93. |
| [48] | Iadanza et al (2020) | inherited retinal diseases, retinitis pigmentosa | Total: 38 patients Evaluation denominator: NR | Reference/comparator: electroretinogram Validation: leave-one-out cross-validation | Accuracy; Sensitivity/recall; Specificity | accuracy 84.6%, sensitivity 93.7%, specificity 78.6% |
| [49] | Ilić et al (2025) | skeletal dysplasias | Total: 45 patients Evaluation denominator: NR | Reference/comparator: molecular diagnosis as the gold standard Validation: physician comparison | Accuracy; Top-k performance | ChatGPT achieved 28 correct diagnoses (62.2%) and DeepSeek correctly identified 29 cases (64.4%), with both models demonstrating comparable performances. The human expert panel achieved a diagnostic top-3 accuracy of 82.2%, significantly surpassing the top-3 accuracy of both LLMs. |
| [50] | Jaroy et al (2024) | Hirschsprung's disease | Total: 178 patients Evaluation denominator: NR | Reference/comparator: rectal biopsy Validation: 5-fold stratified cross-validation | Sensitivity/recall | champion model achieved 100% recall, 35% of HD-negative children spared rectal biopsy, conventional logistic regression missed 40% of HD-positive cases |
| [51] | Song et al (2025) | rare genetic diseases | Total: 500 questions Evaluation denominator: NR | Reference/comparator: HPO Validation: internal validation | Accuracy | RAG LLMs improved diagnostic accuracy, consistency, and reduced temperature-induced variability; Vector RAG outperformed Cypher RAG in most tasks. |
| [52] | Jiang et al (2025) | biliary atresia | Total: 348 patients Evaluation denominator: NR | Reference/comparator: intraoperative cholangiography and histological examination Validation: internal validation and external validation | AUROC/AUC; Accuracy; Sensitivity/recall; Specificity | The combined AI model achieved an AUC of 0.985, sensitivity of 98.2%, specificity of 93.1%, and accuracy of 94.7% in the training cohort, and an AUC of 0.949, sensitivity of 89.8%, specificity of 91.4%, and accuracy of 90.4% in the validation cohort. |
| [53] | Kadi et al (2024) | PAX9, WNT10A, EDA, and other rare genetic dental diseases | Total: 272 dental panoramic X-rays Evaluation denominator: NR | Reference/comparator: genetic analysis, dental age assessment Validation: internal validation, cross-validation | Sensitivity/recall; PPV/precision; F-score | mAP > 79%, precision and recall on test data, F1-score of 92.47% on control images, 69.41% on agenesis dataset |
| [54] | Kauffman et al (2025) | neonatal culture-negative sepsis, postoperative acute kidney injury | Total: 8,015 patients Evaluation denominator: NR | Reference/comparator: physician-provided heuristics, clinical guidelines Validation: internal validation, external validation | Rule-in/rule-out potential | InfEHR outperformed clinician-provided heuristics with superior rule-in and rule-out potentials in both PO-AKI and CN-S task settings. The resulting rule-in potentials for CN-S were 16.018 (Train) and 12.49 (Val) compared to 1.013 (clinician-provided) and 2.5 times greater rule-in potentials and 2.13 times greater rule-out potentials for PO-AKI. |
| [55] | Kothari et al (2022) | PTEN hamartoma tumor syndrome (PHTS) | Total: 1,215 patients Evaluation denominator: NR | Reference/comparator: Cleveland Clinic pediatric clinical criteria Validation: internal validation, physician comparison | PPV/precision; Diagnostic yield/rate | The yield of the computational phenotype ranged from 82 to 94% at the three centers. The positive predictive value of this approach exceeded 80%. |
| [56] | Li et al (2026) | biliary atresia | Total: 50 patients Evaluation denominator: NR | Reference/comparator: intraoperative cholangiography and histopathology Validation: internal validation with repeated stratified 5-fold cross-validation | AUROC/AUC; Sensitivity/recall; Specificity; F-score | The logistic regression model achieved a mean AUC of 0.978 +/- 0.047, sensitivity 0.955 +/- 0.096, specificity 0.922 +/- 0.134, and F1-score 0.949 +/- 0.072. The random forest model had a mean AUC of 0.951 +/- 0.087, sensitivity 0.949 +/- 0.096, specificity 0.850 +/- 0.161, and F1-score 0.923 +/- 0.071. |
| [57] | Li et al (2026) | eosinophilic esophagitis | Total: NR Evaluation denominator: NR | Reference/comparator: histopathological analysis Validation: internal validation | AUROC/AUC | The study identified 54 differentially expressed genes associated with eosinophilic esophagitis, including CTSG and IL1RL1, which were used to develop a machine learning model with high diagnostic performance (AUC=0.972). |
| [58] | Li et al (2025) | inborn errors of metabolism (IEMs) | Total: 355,197 cases Evaluation denominator: NR | Reference/comparator: expert-reported IEMs, including mild conditions Validation: internal validation, 100 repetitions with 95% confidence interval | Accuracy; Sensitivity/recall; Specificity; F-score | First-stage model: sensitivity 0.938 (95% CI = 0.937-0.939), F1-value 0.847 (95% CI = 0.845-0.849). Second-stage models: average sensitivity 0.992, average specificity 0.944, average accuracy 0.969. |
| [59] | Ma et al (2025) | biliary atresia | Total: 681 BA patients, 898 non-BA patients Evaluation denominator: NR | Reference/comparator: surgical exploration and intraoperative cholangiogram Validation: internal validation, external validation | AUROC/AUC; Accuracy; Sensitivity/recall; Specificity; PPV/precision; NPV | The multimodal model achieved an AUC of 0.9870, a sensitivity of 0.9561, a specificity of 0.9630, an accuracy of 0.9600, a positive predictive value of 0.9515, and a negative predictive value of 0.9665 on the internal test set. On the external test set, the multimodal model achieved an AUC of 0.9740, a sensitivity of 0.9130, a specificity of 0.9510, an accuracy of 0.9357, a positive predictive value of 0.9515, and a negative predictive value of 0.9665. |
| [60] | Mao et al (2025) | rare diseases | Total: 2,271 cases Evaluation denominator: NR | Reference/comparator: human expert diagnoses Validation: internal validation, external validation, physician comparison | Sensitivity/recall; Top-k performance; Rank | PhenoBrain achieved an average top-3 recall of 0.513 and a top-10 recall of 0.654, outperforming human experts and large language models. The ensemble method of PhenoBrain achieved an average median rank of 3.3, a top-3 recall of 0.513, and a top-10 recall of 0.654. |
| [61] | Méndez Barrera et al (2023) | primary immune deficiencies | Total: 2,396 patients Evaluation denominator: NR | Reference/comparator: gold standard diagnosis Validation: internal validation with random train/test split | AUROC/AUC; Accuracy | Accuracy: 0.70-0.80 for most diseases; AUC: 0.70-0.80; Gini indexes: 0.50; SHAP values show feature importance for prediction model |
| [62] | Mohammed et al (2026) | biliary atresia | Total: 1,439 patients, 4,546 sonographic gallbladder images Evaluation denominator: NR | Reference/comparator: intra-operative cholangiography, US-guided cholecystocholangiography, biopsy, or follow-up Validation: internal validation, external validation | AUROC/AUC | AUC 0.9547 (image-level), AUC 0.9636 (patient-level), outperformed individual deep learning models and other ensemble methods |
| [63] | Orenbuch et al (2025) | severe developmental disorders | Total: 31,058 cases Evaluation denominator: NR | Reference/comparator: ClinVar, UK Biobank Validation: internal validation, external validation | AUROC/AUC; Enrichment | popEVE achieved state-of-the-art performance in variant effect prediction, with AUC of 0.97 in distinguishing diagnosed cases from controls, and 15-fold enrichment of severely deleterious variants in SDD cases. |
| [64] | Porfireva et al (2025) | primary immunodeficiencies | Total: 4,825 missense variants Evaluation denominator: NR | Reference/comparator: gold standard diagnosis Validation: leave-one-out cross-validation, 5-fold cross-validation | AUROC/AUC; Balanced accuracy; F-score; MCC | AUC 0.831 +/- 0.037, Balanced accuracy 0.763 +/- 0.034, MCC 0.457 +/- 0.06, F-measure 0.623 +/- 0.07 |
| [65] | Porras et al (2021) | genetic syndromes | Total: 2,800 facial photographs Evaluation denominator: NR | Reference/comparator: clinical or molecular diagnosis Validation: cross-validation | Accuracy; Sensitivity/recall; Specificity | 88% accuracy, 90% sensitivity, 86% specificity in total population; 90% accuracy in White and Hispanic populations; 82% or higher in all racial and ethnic groups |
| [66] | Rassmann et al (2023) | skeletal dysplasias (achondroplasia, hypochondroplasia, pseudohypoparathyroidism, Noonan, Silver-Russell, Ullrich-Turner syndromes) | Total: 568 radiographs from 189 patients (GDBD); RSNA dataset 12,611 training and 200 test images Evaluation denominator: 200 test images | Reference/comparator: manual bone age ratings by expert radiologists Validation: internal validation on RSNA test set, external validation on GDBD and DHA cohorts | Accuracy; PPV/precision; MAE/MAD/RMSE | RSNA test set: MAD 3.87 months; GDBD dysplastic cohort: MAD 5.84 months; DHA external cohort: MAD 5.81 months, RMSE 7.67 months, 1-year accuracy 92.9%; test-retest precision 2.74 months on longitudinal data. |
| [67] | Reiley et al (2023) | Mendelian disorders | Total: 25 trios Evaluation denominator: NR | Reference/comparator: RapSeq pipeline Validation: internal validation | Top-k performance; Rank | Dx29 ranked the correct diagnosis in the top 5 diseases in 88% of cases and in the top 10 diseases in 92% of cases. |
| [68] | Rider et al (2021) | primary immunodeficiency diseases | Total: 1,762 patients Evaluation denominator: NR | Reference/comparator: International Union of Immunological Societies (IUIS) diagnostic categories Validation: internal validation and external validation | AUROC/AUC; Accuracy | The Bayesian network model (PI Prob) achieved an area under the receiver operator characteristic curve (AUROC) of 0.945 (p < 0.0001) and was 89% accurate in classifying patients with primary immunodeficiency diseases. |
| [69] | Robinson et al (2020) | juvenile-onset systemic lupus erythematosus | Total: 67 patients, 39 healthy controls Evaluation denominator: NR | Reference/comparator: American College of Rheumatology revised classification criteria for lupus Validation: ten-fold cross-validation | Accuracy; Rank | The BRF model discriminated patients with juvenile-onset SLE from healthy controls with 90.9% prediction accuracy. The top-ranked immunological features from the BRF model were confirmed using sPLS-DA and logistic regression, and included total CD4, total CD8, CD8 effector memory, and CD8 naive T cells, Bm1, and unswitched memory B cells, total CD14 monocytes, and invariant natural killer T cells. |
| [70] | Rudy et al (2025) | rare genetic disorders | Total: 19 patients Evaluation denominator: NR | Reference/comparator: clinical evaluation and genetic testing Validation: physician comparison | Case match rate | Face2Gene suggested a diagnosis in 17 out of 19 patients, with a high Gestalt meter score in 14 patients. The software suggested a specific syndrome in 15 out of 17 patients, and the diagnosis was confirmed in all cases. |
| [71] | Salvia et al (2025) | rare diseases, including Gaucher, ALS, and AADC deficiency | Total: 170,000+ patients Evaluation denominator: NR | Reference/comparator: clinical diagnosis Validation: internal validation, external validation | AUROC/AUC; F-score | The HTT model achieved an F1-score of 0.69, AUC of 0.89, and detected diseases up to 12 days in advance of clinical detection. |
| [72] | Schmidt et al (2024) | ultrarare genetic disorders | Total: 1,577 patients Evaluation denominator: NR | Reference/comparator: gold standard molecular diagnosis by multidisciplinary teams Validation: prospective national cohort, internal validation with held-out test set | AUROC/AUC; Diagnostic yield/rate | Molecular diagnosis established in 32% of patients (370 distinct causes); 34 novel and 23 candidate genotype-phenotype associations identified; YieldPred AUC 0.67 (95% CI 0.61-0.74); facial image analysis with GestaltMatcher improved variant prioritization efficiency. |
| [73] | Schwitzkowski et al (2026) | inborn errors of immunity/primary immunodeficiency (IEI/PID) | Total: 825 patients Evaluation denominator: NR | Reference/comparator: gold standard diagnosis Validation: internal validation, cross-validation | Balanced accuracy; Top-k performance | Balanced accuracy: 44% for IUIS categories, 73% for monogenetic defects; Top 3 accuracy: 86% for IUIS categories, 93% for monogenetic defects |
| [74] | Song et al (2025) | rare genetic diseases | Total: 509 publications, 1,147 data records Evaluation denominator: NR | Reference/comparator: gold standard diagnosis Validation: internal validation, cross-validation | Accuracy; F-score; Top-k performance | 80.19% Top-1 accuracy, 0.59 Macro-F1 score |
| [75] | Ustach et al (2026) | Mendelian disorders | Total: 9,989 cases Evaluation denominator: NR | Reference/comparator: standard clinical analysis Validation: internal validation | Sensitivity/recall; Top-k performance; Rank | Multiscore prioritized the reported positive gene with a median rank of 3 and mean rank of 6.35. The average recall (sensitivity) of Multiscore was 33% in the top 1, 69% in the top 5, 83% in the top 10, and 93% in the top 20 ranked genes. |
| [76] | van der Donk et al (2019) | rare genomic neurodevelopmental disorders, intellectual disability | Total: 71 patients with Koolen-de Vries syndrome, 14 patients with PACS1, 11 patients with PPM1D, 16 patients with PHIP, and 71-80 matched ID controls Evaluation denominator: NR | Reference/comparator: gold standard diagnosis based on clinical evaluation Validation: internal validation and cross-validation | CIF | The hybrid model achieved a clustering improvement factor (CIF) of 1.191 for Koolen-de Vries syndrome, 2.523 for PACS1, 1.713 for PPM1D, and 2.239 for PHIP, indicating significant facial similarity between patients with the same syndrome. |
| [77] | Vargova et al (2025) | Hirschsprung disease | Total: 218 patients Evaluation denominator: NR | Reference/comparator: rectal biopsy Validation: internal validation | Balanced accuracy; Sensitivity/recall; Specificity; NPV | The model achieved a sensitivity of 81.8%, specificity of 93.0%, and balanced accuracy of 0.874. The combined model integrating image and clinical data achieved a sensitivity of 90.9%, specificity of 93.0%, and negative predictive value of 97.6%. |
| [78] | Wang et al (2026) | rare diseases | Total: NR patients Evaluation denominator: NR | Reference/comparator: gold standard diagnosis Validation: internal validation | Accuracy; Sensitivity/recall; F-score; Top-k performance | The system received a comprehensive score of 0.72 out of 1.0 from an automated static code analysis and evaluation system. It demonstrated strong performance in functional completeness (0.83) and clinical accuracy (0.79). The proposed system demonstrates superior capability in discriminating rare diseases compared to the control group (with an accuracy of 0.844 1 and F1-score of 0.601 4), along with a relatively strong performance in rare disease categorization (with a Top-3 recall rate of 0.550 3). |
| [79] | Wang and Lü (2020) | Rare genetic diseases | Total: 1,100 patients Evaluation denominator: NR | Reference/comparator: OMIM, Orphanet, and DECIPHER Validation: Internal validation | Prediction-change outcome | The algorithm showed good results on the simulated data set, with 2.8% of predictions improved and 97.2% remaining unchanged. |
| [80] | Xie et al (2025) | inherited metabolic disorders (IMDs) | Total: 119 patients Evaluation denominator: NR | Reference/comparator: gold standard diagnosis by California NBS program Validation: internal validation | Sensitivity/recall; False-positive outcome | AI/ML-based analysis achieved 94.3% sensitivity for confirming true positives, while reducing false positives by 100% for GA-I and OTCD, 54.8% for PA/MMA, and 0% for VLCADD. Metabolomics-AI/ML-enhanced analysis reduced false positives by 80% for GA-I, 77.4% for PA/MMA, 100% for OTCD, and 41.4% for VLCADD. |
| [81] | Yahya et al (2025) | genetic disorders, dysmorphic syndromes | Total: 151 patients Evaluation denominator: NR | Reference/comparator: gold standard genetic diagnosis Validation: internal validation | Top-k performance | 56% of patients had correct diagnosis in top 3 suggestions, 47% with facial analysis only, p = 0.001 |
| [82] | Yang et al (2021) | Inherited Metabolic Diseases | Total: 5,907,547 Evaluation denominator: NR | Reference/comparator: Clinical Diagnosis Validation: Internal Validation, Physician Comparison | Sensitivity/recall; PPV/precision; False-positive outcome | The model achieved a sensitivity of 0.74% and a positive predictive value of 2.66% compared to clinicians. The model reduced the number of false positives by 36.89% compared to clinicians. |
| [83] | Yang et al (2024) | inherited metabolic diseases | Total: 309,102 patients Evaluation denominator: NR | Reference/comparator: next-generation sequencing (NGS) Validation: internal validation | Sensitivity/recall; Specificity; PPV/precision; NPV | The ML system achieved a sensitivity of 93.42% and a specificity of 78.57%, surpassing the performance of the reference interval. The ML system demonstrated increased positive predictive value (PPV) and negative predictive value (NPV). |
| [84] | Yang et al (2022) | Noonan syndrome | Total: 189 patients, 162 NS cases, 16,200 controls Evaluation denominator: NR | Reference/comparator: gold standard diagnosis Validation: internal validation, external validation | AUPRC/PR-AUC; Sensitivity/recall; PPV/precision; F-score | CNN model achieved PRAUC 0.43, precision 0.47, recall 0.52, F1 score 0.494 in test set 1; PRAUC 0.16, precision 0.33, recall 0.40, F1 score 0.362 in test set 2 |
| [85] | Yates et al (2021) | rare diseases | Total: 1,042 patients Evaluation denominator: NR | Reference/comparator: Human Phenotype Ontology terms Validation: internal validation, 10-fold cross-validation | Balanced accuracy | 4 statistically significant clusters identified, 2 clusters characterized by growth disorders, 2 clusters by neurodevelopmental symptoms; support vector classifier showed 0.89 balanced accuracy on test data |
| [86] | Young et al (2024) | rare pediatric genetic diseases | Total: 61 cases Evaluation denominator: NR | Reference/comparator: physician diagnosis Validation: Fisher's exact test | Accuracy | Diagnostic accuracy: GPT-4 13.1%, GPT-4 HPO 8.2%, Gemini Pro 8.2%. GPT-4 HPO outperformed other models in identifying diagnosis among differential list and correct disease category. |
| [87] | Yu et al (2024) | DiGeorge syndrome, Down syndrome, Williams syndrome | Total: 109 patients Evaluation denominator: NR | Reference/comparator: NIH data Validation: 10-fold cross-validation | AUROC/AUC; Accuracy; Sensitivity/recall | The model achieved an AUC of 0.84, sensitivity of 0.87, and accuracy of 0.91. The fusion of geometric features and deep learning features improved the performance of the model. |
| [88] | Zaunseder et al (2024) | Glutaric aciduria type 1 | Total: 1,025,953 newborns Evaluation denominator: NR | Reference/comparator: gold standard diagnosis Validation: internal validation, cross-validation | False-positive outcome | The digital-tier strategy reduced the false-positive rate by over 90% compared to regular NBS while identifying all confirmed individuals with GA1 correctly. The best-performing algorithm, logistic regression, reduced the number of false positives by 93.61% on the full data set and by 95.05% on the suspected diagnosis data set. |
| [89] | Zhang et al (2016) | Duchenne muscular dystrophy | Total: 30 participants; 485 MRI images Evaluation denominator: NR | Reference/comparator: Muscle-biopsy pathological diagnosis for DMD; clinically healthy controls Validation: Internal validation using 10-fold cross-validation | Accuracy; Sensitivity/recall; Specificity | The best T1-weighted MRI SVM classifier at wavelet decomposition level 2 (C=1, gamma=0.083) achieved 96.9% sensitivity, 97.3% specificity, and 97.1% accuracy. |
| [90] | Zhao et al (2026) | rare diseases (2,919 diseases spanning 14 medical specialties) | Total: 6,401 clinical cases covering 2,919 rare diseases Evaluation denominator: NR | Reference/comparator: gold standard diagnosis; compared with 15+ methods including Exomiser and LLMs Validation: internal validation, external validation across nine datasets | Sensitivity/recall; Agreement | Average Recall@1 57.18% on HPO-based tasks (23.79% higher than next best method); 69.1% Recall@1 on multimodal tests vs Exomiser 55.9% on 168 cases; 95.4% expert agreement on reasoning chains. |
| [91] | Zheng et al (2026) | growth hormone deficiency, idiopathic central precocious puberty | Total: 274 patients Evaluation denominator: NR | Reference/comparator: hormone stimulation tests Validation: internal validation, cross-validation | Dice coefficient | ADHTransNet achieved a mean Dice coefficient of 0.8915 +/- 0.0360, and significant correlations were observed between true hormone levels and predicted values in the GHD group (r = 0.359, p < 0.001; r = 0.422, p < 0.001). |
| [92] | Zucca et al (2024) | genetic diseases | Total: 35 patients Evaluation denominator: NR | Reference/comparator: ACMG/AMP guidelines Validation: internal validation, Leave-One-Proband-Out Cross Validation | Rank | The model ranked the causative variant in the top 5th positions in 76% of cases, and in the top 10th positions in 100% of cases. The phenotypic similarity score showed a better performance in terms of ranking of the causative gene compared to other published metrics. |

Abbreviations: AUC, area under the curve; AUPRC, area under the precision-recall curve; AUROC, area under the receiver operating characteristic curve; CIF, clustering improvement factor; DL, deep learning; EHR, electronic health record; F-score, F measure; LLM, large language model; MAE, mean absolute error; MAD, mean absolute deviation; MCC, Matthews correlation coefficient; ML, machine learning; NPV, negative predictive value; NR, not reported; PPV, positive predictive value; RMSE, root-mean-square error. Explicitly identifiable evaluation denominators were available for 1 of 81 studies. NR indicates that the article did not clearly report a separate evaluable denominator for the specific performance metric. When a separate test, validation, or external evaluation denominator was unavailable, the total study sample size and validation design were retained to contextualize the reported performance.

# References

12. Şahin A, Sonmez G, Unsal H, Cagdas D. Machine learning‐based prediction of inborn errors of immunity in patients with low IgE levels. Pediatric Allergy and Immunology. 2026;37(1). doi: 10.1111/pai.70283.

13. Alsentzer E, Li MM, Kobren SN, Noori A, Kohane IS, Zitnik M. Few shot learning for phenotype-driven diagnosis of patients with rare genetic diseases. npj Digital Medicine. 2025;8(1). doi: 10.1038/s41746-025-01749-1.

14. Andriessen E, de Boer E, Lyon GJ, de Vries BBA, Ockeloen CW, Dingemans AJM. Functional Data Strengthen Clinical Validation of PhenoScore Phenotype-Guided AI for ANKRD11 Missense Variants. Clinical Genetics. 2026;109(6):1038-48. doi: 10.1111/cge.70159.

15. Aref-Eshghi E, Rodenhiser DI, Schenkel LC, Lin H, Skinner C, Ainsworth P, et al. Genomic DNA Methylation Signatures Enable Concurrent Diagnosis and Clinical Genetic Variant Classification in Neurodevelopmental Syndromes. The American Journal of Human Genetics. 2018;102(1):156-74. doi: 10.1016/j.ajhg.2017.12.008.

16. Benichou L, Breton L, Garcelon N, Benichou B, Lienhard O, Amiel J, et al. Evaluation of a New Inclusive Next‐Generation Synthetic Face Tool for Dysmorphology. American Journal of Medical Genetics Part A. 2025;200(3):685-94. doi: 10.1002/ajmg.a.64298.

17. Bolmer E, Schmidt P, Fischer I, Rassmann S, Ruder A, Hustinx A, et al. Bone2Gene: Next-generation Phenotyping of Rare Bone Diseases. 2026. doi: 10.64898/2026.03.25.26349289.

18. Burns G, Kauffman C, Manion M, Pai R-A, Milla C, O’Connor MG, et al. Feasibility of machine learning analysis for the identification of patients with possible primary ciliary dyskinesia. Orphanet Journal of Rare Diseases. 2025;20(1). doi: 10.1186/s13023-025-03966-z.

19. Cao L, Huang YS, Wu C, Getz K, Miller TP, Ruiz J, et al. Leveraging machine learning to identify acute myeloid leukemia patients and their chemotherapy regimens in an administrative database. Pediatric Blood Cancer. 2023;70(5). doi: 10.1002/pbc.30260.

20. Chen F, Ahimaz P, Nguyen QM, Lewis R, Chung WK, Ta CN, et al. Phenotype driven molecular genetic test recommendation for diagnosing pediatric rare disorders. npj Digital Medicine. 2024;7(1). doi: 10.1038/s41746-024-01331-1.

21. Chen H, Zhao Z, Zhou S, Hu S, Wang J, Jin Y, et al. RareArena: a comprehensive benchmark dataset unveiling the potential of large language models in rare disease diagnosis. The Lancet Digital Health. 2026;8(2):100953. doi: 10.1016/j.landig.2025.100953.

22. Chen X, Faviez C, Vincent M, Briseño-Roa L, Faour H, Annereau J-P, et al. Patient-Patient Similarity-Based Screening of a Clinical Data Warehouse to Support Ciliopathy Diagnosis. Frontiers in Pharmacology. 2022;13. doi: 10.3389/fphar.2022.786710.

23. Chen S, Zhao X, Fu H, Huang J, Wu Z, Dashtbozorg B, et al. SABPI-Net: A Structure-Aware Bidirectional Proxy Interaction Network for Infantile Retinal Disease Diagnosis. IEEE Transactions on Medical Imaging. 2026;45(5):2229-43. doi: 10.1109/tmi.2026.3650968.

24. Chen Y, He Z, Yang KG, Qin X, Lau AY-C, Liu Z, et al. Identification of Atypical Scoliosis Patterns Using X-ray Images Based on Fine-Grained Techniques in Deep Learning. Global Spine Journal. 2025;16(1):501-12. doi: 10.1177/21925682251349999.

25. Cohen ASA, Farrow EG, Abdelmoity AT, Alaimo JT, Amudhavalli SM, Anderson JT, et al. Genomic answers for children: Dynamic analyses of >1000 pediatric rare disease genomes. Genetics in Medicine. 2022;24(6):1336-48. doi: 10.1016/j.gim.2022.02.007.

26. Crisafulli S, Ingrasciotta Y, Vitturi G, Fontana A, L’Abbate L, Alessi Y, et al. Epidemiological analysis to identify predictors of X-linked hypophosphatemia (XLH) diagnosis in an Italian pediatric population: the EPIX project. Endocrine. 2024;85(2):894-905. doi: 10.1007/s12020-024-03793-5.

27. Cuce F, Tulum G, Işık MI, Baştemur M, Arslan M, Ünay B, et al. Diagnostic value of machine-learning using conventional magnetic resonance imaging markers for pediatric idiopathic intracranial hypertension: a retrospective study. Pediatric Radiology. 2026. doi: 10.1007/s00247-026-06638-7.

28. Spencer DA, Liu X, Mosema-Be-Amoti K, Kandosi G, Bramble MS, Al Munajjed F, et al. Identification and Developmental Analysis of the Facial Characteristics Associated with Sickle Cell Disease using Machine Learning. 2026. doi: 10.64898/2026.03.03.26346563.

29. De Furia A, Branco P, Henderson M. Enhancing TSH-based congenital hypothyroidism screening using machine learning and resampling algorithms. BMC Medical Informatics and Decision Making. 2025;25(1). doi: 10.1186/s12911-025-03312-0.

30. De La Vega FM, Chowdhury S, Moore B, Frise E, McCarthy J, Hernandez EJ, et al. Artificial intelligence enables comprehensive genome interpretation and nomination of candidate diagnoses for rare genetic diseases. Genome Medicine. 2021;13(1). doi: 10.1186/s13073-021-00965-0.

31. del Rincón J, Gil-Salvador M, Lucia-Campos C, Acero L, Trujillano L, Arnedo M, et al. AI-Based Facial Phenotyping Supports a Shared Molecular Axis in PACS1-, PACS2-, and WDR37-Related Syndromes. International Journal of Molecular Sciences. 2025;26(16):7964. doi: 10.3390/ijms26167964.

32. Dingemans AJM, Hinne M, Truijen KMG, Goltstein L, van Reeuwijk J, de Leeuw N, et al. PhenoScore quantifies phenotypic variation for rare genetic diseases by combining facial analysis with other clinical features using a machine-learning framework. Nature Genetics. 2023;55(9):1598-607. doi: 10.1038/s41588-023-01469-w.

33. Echeverry-Quiceno LM, Candelo E, Gómez E, Solís P, Ramírez D, Ortiz D, et al. Population-specific facial traits and diagnosis accuracy of genetic and rare diseases in an admixed Colombian population. Scientific Reports. 2023;13(1). doi: 10.1038/s41598-023-33374-x.

34. Elmas M, Gogus B. Success of Face Analysis Technology in Rare Genetic Diseases Diagnosed by Whole-Exome Sequencing: A Single-Center Experience. Molecular Syndromology. 2020;11(1):4-14. doi: 10.1159/000505800.

35. Fadul R, Tumzghi N, Seghier M, Al-Jasmi F, AlShehhi A. Explainable AI-driven graph-based neural networks for mucopolysaccharidoses diagnosis. BioData Mining. 2026;19(1). doi: 10.1186/s13040-026-00523-7.

36. Feng J, Liu Z, Shen L, Luo X, Chen Y, Li L, et al. IESS-FusionNet: Physiologically Inspired EEG-EMG Fusion with Linear Recurrent Attention for Infantile Epileptic Spasms Syndrome Detection. Bioengineering. 2025;13(1):57. doi: 10.3390/bioengineering13010057.

37. Garcelon N, Neuraz A, Salomon R, Bahi-Buisson N, Amiel J, Picard C, et al. Next generation phenotyping using narrative reports in a rare disease clinical data warehouse. Orphanet Journal of Rare Diseases. 2018;13(1). doi: 10.1186/s13023-018-0830-6.

38. Greco KF, Yang Z, Li M, Tong H, Morini Sweet S, Geva A, et al. A weakly supervised transformer for rare disease diagnosis and subphenotyping from EHRs with pulmonary case studies. npj Digital Medicine. 2026;9(1). doi: 10.1038/s41746-026-02406-x.

39. Groen J, de Haan BM, Overduin RJ, Haijer-Schreuder AB, Derks TGJ, Heiner-Fokkema MR. A machine learning model accurately identifies glycogen storage disease Ia patients based on plasma acylcarnitine profiles. Orphanet Journal of Rare Diseases. 2025;20(1). doi: 10.1186/s13023-025-03537-2.

40. Hack JB, Watkins JC, Hammer MF. Machine learning models reveal distinct disease subgroups and improve diagnostic and prognostic accuracy for individuals with pathogenic gain-of-function variants. Biology Open. 2024;13(4). doi: 10.1242/bio.060286.

41. Hennocq Q, Bongibault T, Bizière M, Delassus O, Douillet M, Cormier‐Daire V, et al. An automatic facial landmarking for children with rare diseases. American Journal of Medical Genetics Part A. 2023;191(5):1210-21. doi: 10.1002/ajmg.a.63126.

42. Herr K, Lu P, Diamreyan K, Xu H, Mendonca E, Weaver KN, et al. Estimating prevalence of rare genetic disease diagnoses using electronic health records in a children’s hospital. Human Genetics and Genomics Advances. 2024;5(4):100341. doi: 10.1016/j.xhgg.2024.100341.

43. Hinterwimmer F, Consalvo S, Neumann J, Micheler C, Wilhelm N, Lang J, et al. From Self-supervised Learning to Transfer Learning with Musculoskeletal Radiographs. Current Directions in Biomedical Engineering. 2022;8(2):9-12. doi: 10.1515/cdbme-2022-1003.

44. Hong D, Zheng Y-Y, Xin Y, Sun L, Yang H, Lin M-Y, et al. Genetic syndromes screening by facial recognition technology: VGG-16 screening model construction and evaluation. Orphanet Journal of Rare Diseases. 2021;16(1). doi: 10.1186/s13023-021-01979-y.

45. Hsieh T-C, Bar-Haim A, Moosa S, Ehmke N, Gripp KW, Pantel JT, et al. GestaltMatcher facilitates rare disease matching using facial phenotype descriptors. Nature Genetics. 2022;54(3):349-57. doi: 10.1038/s41588-021-01010-x.

46. Huang P, Huang J, Huang Y, Yang M, Kong R, Sun H, et al. Optimization and evaluation of facial recognition models for Williams-Beuren syndrome. European Journal of Pediatrics. 2024;183(9):3797-808. doi: 10.1007/s00431-024-05646-9.

47. Huang SD, Bamba V, Bothwell S, Fechner PY, Furniss A, Ikomi C, et al. Development and validation of a computable phenotype for Turner syndrome utilizing electronic health records from a national pediatric network. American Journal of Medical Genetics Part A. 2023;194(4). doi: 10.1002/ajmg.a.63495.

48. Iadanza E, Goretti F, Sorelli M, Melillo P, Pecchia L, Simonelli F, et al. Automatic Detection of Genetic Diseases in Pediatric Age Using Pupillometry. IEEE Access. 2020;8:34949-61. doi: 10.1109/access.2020.2973747.

49. Ilić N, Marić N, Cvetković D, Bogosavljević M, Bukara-Radujković G, Krstić J, et al. The Artificial Intelligence-Assisted Diagnosis of Skeletal Dysplasias in Pediatric Patients: A Comparative Benchmark Study of Large Language Models and a Clinical Expert Group. Genes. 2025;16(7):762. doi: 10.3390/genes16070762.

50. G. Jaroy E, Risa GT, Farstad IN, Emblem R, Ougland R. A recall-optimised machine learning framework for small data improves risk stratification for Hirschsprung's disease. Informatics in Medicine Unlocked. 2024;48:101530. doi: 10.1016/j.imu.2024.101530.

51. Song J, Xu Z, He M, Feng J, Shen B. Graph retrieval augmented large language models for facial phenotype associated rare genetic disease. npj Digital Medicine. 2025;8(1). doi: 10.1038/s41746-025-01955-x.

52. Jiang J-Y, Dong R, Sun Y-H, Yang Y-F, Verkade HJ, Cai X, et al. Development and validation of a minimally invasive diagnostic model for biliary atresia using artificial intelligence. World Journal of Pediatrics. 2025;21(12):1289-98. doi: 10.1007/s12519-025-00988-2.

53. Kadi H, Kawczynski M, Bendjama S, Flores JZ, Leong-Hoi A, de Lastic H, et al. i-Dent: A virtual assistant to diagnose rare genetic dental diseases. Computers in Biology and Medicine. 2024;180:108927. doi: 10.1016/j.compbiomed.2024.108927.

54. Kauffman J, Holmes E, Vaid A, Charney A, Kovatch P, Lampert J, et al. InfEHR: Resolving Clinical Uncertainty through Deep Geometric Learning on Electronic Health Records. 2025. doi: 10.1038/s41467-025-63366-6.

55. Kothari C, Srivastava S, Kousa Y, Izem R, Gierdalski M, Kim D, et al. Validation of a computational phenotype for finding patients eligible for genetic testing for pathogenic PTEN variants across three centers. Journal of Neurodevelopmental Disorders. 2022;14(1). doi: 10.1186/s11689-022-09434-0.

56. Li G, Wang D, Sun D, Li S, Hua K, Gu Y, et al. A minimalist and robust diagnostic model for neonatal biliary atresia: Harnessing MMP-7 and machine learning in a time-critical setting. Journal of Pediatric Surgery. 2026;61(7):163090. doi: 10.1016/j.jpedsurg.2026.163090.

57. Li Z, Iliminur A, Li Z, Wu J. Machine learning algorithms integrate single-cell and bulk RNA data to reveal the immuno-molecular characteristics of mast cells in eosinophilic esophagitis. China Medical Herald. 2026;23(10):45-54. doi: 10.20047/j.issn1673-7210.25091014.

58. Li H, Gao S, Wu D, Zhu M, Hu Z, Fang K, et al. Training machine learning models to detect rare inborn errors of metabolism (IEMs) based on GC–MS urinary metabolomics for diseases screening. International Journal of Medical Informatics. 2025;195:105765. doi: 10.1016/j.ijmedinf.2024.105765.

59. Ma Y, Yang Y, Du Y, Jin L, Liang B, Zhang Y, et al. Development of an artificial intelligence-based multimodal diagnostic system for early detection of biliary atresia. BMC Medicine. 2025;23(1). doi: 10.1186/s12916-025-03962-x.

60. Mao X, Huang Y, Jin Y, Wang L, Chen X, Liu H, et al. A phenotype-based AI pipeline outperforms human experts in differentially diagnosing rare diseases using EHRs. npj Digital Medicine. 2025;8(1). doi: 10.1038/s41746-025-01452-1.

61. Méndez Barrera JA, Rocha Guzmán S, Hierro Cascajares E, Garabedian EK, Fuleihan RL, Sullivan KE, et al. Who's your data? Primary immune deficiency differential diagnosis prediction via machine learning and data mining of the USIDNET registry. Clinical Immunology. 2023;255:109759. doi: 10.1016/j.clim.2023.109759.

62. Mohammed AAQ, Geng X, Wang J, Fateh AA, Hassan M, Ali Z. SSL-OHE: A self-supervised ensemble approach for early diagnosis of biliary atresia from sonographic images. Biomedical Signal Processing and Control. 2026;112:108539. doi: 10.1016/j.bspc.2025.108539.

63. Orenbuch R, Shearer CA, Kollasch AW, Spinner AD, Hopf T, van Niekerk L, et al. Proteome-wide model for human disease genetics. Nature Genetics. 2025;57(12):3165-74. doi: 10.1038/s41588-025-02400-1.

64. Porfireva ES, Zadorozhny AD, Rudik AV, Filimonov DA, Lagunin AA. Sequence-structure based prediction of pathogenicity for amino acid substitutions in proteins associated with primary immunodeficiencies. Frontiers in Immunology. 2025;16. doi: 10.3389/fimmu.2025.1492751.

65. Porras AR, Rosenbaum K, Tor-Diez C, Summar M, Linguraru MG. Development and evaluation of a machine learning-based point-of-care screening tool for genetic syndromes in children: a multinational retrospective study. The Lancet Digital Health. 2021;3(10):e635-e43. doi: 10.1016/s2589-7500(21)00137-0.

66. Rassmann S, Keller A, Skaf K, Hustinx A, Gausche R, Ibarra-Arrelano MA, et al. Deeplasia: deep learning for bone age assessment validated on skeletal dysplasias. Pediatric Radiology. 2023;54(1):82-95. doi: 10.1007/s00247-023-05789-1.

67. Reiley J, Botas P, Miller CE, Zhao J, Malone Jenkins S, Best H, et al. Open-Source Artificial Intelligence System Supports Diagnosis of Mendelian Diseases in Acutely Ill Infants. Children. 2023;10(6):991. doi: 10.3390/children10060991.

68. Rider NL, Cahill G, Motazedi T, Wei L, Kurian A, Noroski LM, et al. PI Prob: A risk prediction and clinical guidance system for evaluating patients with recurrent infections. PLOS ONE. 2021;16(2):e0237285. doi: 10.1371/journal.pone.0237285.

69. Robinson GA, Peng J, Dönnes P, Coelewij L, Naja M, Radziszewska A, et al. Disease-associated and patient-specific immune cell signatures in juvenile-onset systemic lupus erythematosus: patient stratification using a machine-learning approach. The Lancet Rheumatology. 2020;2(8):e485-e96. doi: 10.1016/s2665-9913(20)30168-5.

70. Rudy NL, Gomes A, Tkemaladze T, Abdul‐Rahman O, Cratsenberg D, Pascolini G, et al. Artificial Intelligence Software Changes Rare Disease Testing Strategy in Real Time: An International Case Series Using Face2Gene. American Journal of Medical Genetics Part A. 2025;197(11). doi: 10.1002/ajmg.a.64171.

71. Salvia VK, Vasundhara V, Kumari MS, B. Sruthi BS, Nair SP, P. Devasudha PD. Deep Learning-Based Early Detection of Rare Diseases Using Electronic Health Records. Journal of Neonatal Surgery. 2025;14(14S):349-66. doi: 10.63682/jns.v14i14s.3653.

72. Schmidt A, Danyel M, Grundmann K, Brunet T, Klinkhammer H, Hsieh T-C, et al. Next-generation phenotyping integrated in a national framework for patients with ultrarare disorders improves genetic diagnostics and yields new molecular findings. Nature Genetics. 2024;56(8):1644-53. doi: 10.1038/s41588-024-01836-1.

73. Schwitzkowski M, Veeranki SPK, Seidel BN, Kindle G, Rusch S, Abd Elaziz D, et al. Machine learning–assisted diagnosis classification of primary immune dysregulation using IDDA2.1 phenotype profiling. Journal of Allergy and Clinical Immunology. 2026;157(2):470-85. doi: 10.1016/j.jaci.2025.10.022.

74. Song J, He M, Ren S, Shen B. An explainable dataset linking facial phenotypes and genes to rare genetic diseases. Scientific Data. 2025;12(1). doi: 10.1038/s41597-025-04922-z.

75. Ustach VD, Guillen Sacoto MJ, McGee S, Gainullin VG, Arvai K, Begtrup A, et al. Multiscore, a gene ranker powered by artificial intelligence and real-world clinical data, shows high sensitivity for the molecular diagnosis of Mendelian disorders in nearly 10,000 exomes and genomes. Human Genetics. 2026;145(1). doi: 10.1007/s00439-025-02797-z.

76. van der Donk R, Jansen S, Schuurs-Hoeijmakers JHM, Koolen DA, Goltstein LCMJ, Hoischen A, et al. Next-generation phenotyping using computer vision algorithms in rare genomic neurodevelopmental disorders. Genetics in Medicine. 2019;21(8):1719-25. doi: 10.1038/s41436-018-0404-y.

77. Vargova P, Varga M, Izquierdo-Hernández B, Gutierrez-Alonso C, Gonazlez-Esgueda A, Cobos-Hernández MV, et al. Artificial Intelligence Enhances Diagnostic Accuracy of Contrast Enemas in Hirschsprung Disease Compared to Clinical Experts. European Journal of Pediatric Surgery. 2025;36(01):003-12. doi: 10.1055/a-2646-2052.

78. Wang P, Kong H, Yang Y, Wang H, Hu J. Research and implementation of an intelligent rare disease discovery system driven by large language models. China Digital Medicine. 2026;21(3):30-8. doi: 10.3969/j.issn.1673-7571.2026.03.005.

79. Wang P, Lü H. A Semantic Similarity Algorithm Using Opposite Phenotypes for the Auxiliary Diagnosis of Rare Diseases. Genomics and Applied Biology 2020;39(12):5868-73. doi: 10.13417/j.gab.039.005868.

80. Xie Y, Peng G, Tikhonova I, Enns G, Zhao H, Cowan T, et al. Improving newborn screening accuracy through genome sequencing, targeted metabolomics, and machine learning. BMC Medical Genomics. 2025;18(1). doi: 10.1186/s12920-025-02261-x.

81. Yahya D, Stoyanova M, Hachmeriyan M, Levkova M. The application of the facial analysis program Face2Gene in a single genetic counseling center: a retrospective study. Egyptian Pediatric Association Gazette. 2025;73(1). doi: 10.1186/s43054-025-00344-z.

82. Yang RL, Yang YL, Wang T, Xu WZ, Yu G, Yang JB, et al. [Establishment of an auxiliary diagnosis system of newborn screening for inherited metabolic diseases based on artificial intelligence technology and a clinical trial]. Zhonghua er ke za zhi Chinese journal of pediatrics. 2021;59(4):286-93. doi: 10.3760/cma.j.cn112140-20201209-01089.

83. Yang X, Ding S, Zhang J, Hu Z, Zhuang D, Wang F, et al. The significance of machine learning in neonatal screening for inherited metabolic diseases. Frontiers in Pediatrics. 2024;12. doi: 10.3389/fped.2024.1366891.

84. Yang Z, Shikany A, Ni Y, Zhang G, Weaver KN, Chen J. Using deep learning and electronic health records to detect Noonan syndrome in pediatric patients. Genetics in Medicine. 2022;24(11):2329-37. doi: 10.1016/j.gim.2022.08.002.

85. Yates J, Gutiérrez-Sacristán A, Jouhet V, LeBlanc K, Esteves C, DeSain TN, et al. Finding commonalities in rare diseases through the undiagnosed diseases network. Journal of the American Medical Informatics Association. 2021;28(8):1694-702. doi: 10.1093/jamia/ocab050.

86. Young CC, Enichen E, Rivera C, Auger CA, Grant N, Rao A, et al. Diagnostic Accuracy of a Custom Large Language Model on Rare Pediatric Disease Case Reports. American Journal of Medical Genetics Part A. 2024;197(2). doi: 10.1002/ajmg.a.63878.

87. Yu W, Xu Q, Liu L. Study on the method of using facial features to improve the accuracy of rare disease prediction in small sample scenarios. China Digital Medicine 2024;19(10):20-7. doi: 10.3969/j.issn.1673-7571.2024.10.004.

88. Zaunseder E, Teinert J, Boy N, Garbade SF, Haupt S, Feyh P, et al. Digital-Tier Strategy Improves Newborn Screening for Glutaric Aciduria Type 1. International Journal of Neonatal Screening. 2024;10(4):83. doi: 10.3390/ijns10040083.

89. Zhang M, Shen Y, Liu X, Chen Y. Classification of MRI for Duchenne Muscular Dystrophy with Feature-Extracted and Machine Learning Methods. Shanghai Sanda University Research and Development 2016(4):12-9.

90. Zhao W, Wu C, Fan Y, Qiu P, Zhang X, Sun Y, et al. An agentic system for rare disease diagnosis with traceable reasoning. Nature. 2026;651(8106):775-84. doi: 10.1038/s41586-025-10097-9.

91. Zheng Q, Jiang X, Sun J, Song L, Zhang L, Liu J. ADHTransNet-based radiomics on multimodal pituitary MRI for non-invasive hormone prediction in children. Computer Methods and Programs in Biomedicine. 2026;276:109235. doi: 10.1016/j.cmpb.2026.109235.

92. Zucca S, Nicora G, De Paoli F, Carta MG, Bellazzi R, Magni P, et al. An AI-based approach driven by genotypes and phenotypes to uplift the diagnostic yield of genetic diseases. Human Genetics. 2024;144(2-3):159-71. doi: 10.1007/s00439-023-02638-x.
