# Supplementary material for: Machine Learning, Large Language Models, and Multimodal AI for Diagnosing Pediatric Rare Diseases: Scoping Review
Source: J Med Internet Res. 2026 Aug 28;28:e96169. doi: 10.2196/96169 (PMC13524366; doi:10.2196/96169)
Supplement: Supplementary file 3 [file jmir-v28-e96169-s003.docx]

**PRISMA-ScR Reporting Checklist**

*Machine Learning, Large Language Models, and Multimodal AI for Diagnosing Pediatric Rare Diseases: A Scoping Review*

Checklist source: PRISMA Extension for Scoping Reviews (PRISMA-ScR). Locations refer to the latest revised manuscript and multimedia appendices submitted with this revision.

| **Section and Topic** | **Item #** | **Checklist item** | **Location where item is reported** |
| --- | --- | --- | --- |
| **TITLE** |  | **TITLE** |  |
| Title | 1 | Identify the report as a scoping review. | Title. |
| **ABSTRACT** |  | **ABSTRACT** |  |
| Structured summary | 2 | Provide a structured summary that includes background, objectives, eligibility criteria, sources of evidence, charting methods, results, and conclusions related to the review question. | Structured Abstract: Background, Objective, Methods, Results, Conclusions. |
| **INTRODUCTION** |  | **INTRODUCTION** |  |
| Rationale | 3 | Describe the rationale for the review in the context of what is already known. | Introduction: paragraphs describing pediatric rare-disease diagnostic delay, AI diagnostic approaches, pediatric-specific challenges, and gaps in existing reviews. |
| Objectives | 4 | Provide an explicit statement of the questions and objectives being addressed with reference to their key elements, such as population or participants, concepts, and context. | Introduction: final paragraph; Methods: Study Design, Review Question, and Reporting Framework. |
| **METHODS** |  | **METHODS** |  |
| Protocol and registration | 5 | Indicate whether a review protocol exists; state if and where it can be accessed, including registration information if available. | Methods: Protocol, Registration, and Amendments; PROSPERO CRD420261326146, Version 1.0 and amended Version 1.1; Multimedia Appendix 1. |
| Eligibility criteria | 6 | Specify characteristics of the sources of evidence used as eligibility criteria and provide a rationale. | Methods: Eligibility Criteria, including Population, Concept, Context, inclusion criteria, exclusion criteria, and rare-disease definition rules. |
| Information sources | 7 | Describe all information sources used in the search and the date of the last search. | Methods: Information Sources and Search Strategy; Results: Study Selection; Figure 1; Multimedia Appendix 1. |
| Search | 8 | Present the full electronic search strategy for at least one database, including any limits used, such that it could be repeated. | Methods: Information Sources and Search Strategy; Multimedia Appendix 1, Full Search Strategy, including source-specific strategies, execution dates, limits, and record counts. |
| Selection of sources of evidence | 9 | State the process for selecting sources of evidence, including title and abstract screening and full-text assessment. | Methods: Study Selection; Results: Study Selection; Figure 1. The manuscript reports independent manual screening, disagreement counts, and third-reviewer adjudication counts. |
| Data charting process | 10 | Describe the methods of charting data from included sources and any processes for obtaining and confirming data. | Methods: Data Charting Process; Multimedia Appendix 2, Tables S1 and S2. |
| Data items | 11 | List and define all variables for which data were sought and any assumptions or simplifications made. | Methods: Data Items and Classification Framework; Multimedia Appendix 2, Tables S1 and S2. |
| Critical appraisal of individual sources of evidence | 12 | If done, provide a rationale for conducting critical appraisal, describe the methods used, and explain how this information was used. | Methods: Critical Appraisal and Evidence Maturity; Results: Validation Strategies and Evidence Maturity; Figure 5. Formal risk-of-bias appraisal was not performed; evidence maturity and validation indicators were charted descriptively. |
| Synthesis of results | 13 | Describe the methods of handling and summarizing the charted data. | Methods: Data Synthesis and Presentation; Table 1; Multimedia Appendix 2, Table S2. |
| **RESULTS** |  | **RESULTS** |  |
| Selection of sources of evidence | 14 | Give numbers of sources screened, assessed for eligibility, and included in the review, with reasons for exclusions at each stage, ideally using a flow diagram. | Results: Study Selection; Figure 1. |
| Characteristics of sources of evidence | 15 | For each source of evidence, present characteristics for which data were charted and provide citations. | Results: Included Studies and Characteristics of Included Studies; Figures 2 and 3; Multimedia Appendix 2, Table S1. |
| Critical appraisal within sources of evidence | 16 | If done, present data on critical appraisal of included sources of evidence. | Results: Validation Strategies and Evidence Maturity; Figure 5. Formal risk-of-bias appraisal was not conducted. |
| Results of individual sources of evidence | 17 | For each included source of evidence, present the relevant charted data related to the review questions and objectives. | Results: Task-Specific Performance Findings; Multimedia Appendix 2, Tables S1 and S2. |
| Synthesis of results | 18 | Summarize and present the charted data in relation to the review questions and objectives. | Results: Artificial Intelligence Technologies and Input Modalities; Diagnostic Tasks; Validation Strategies and Evidence Maturity; Task-Specific Performance Findings; Integrated Evidence Synthesis; Table 1; Figures 4 and 5; Multimedia Appendix 2. |
| **DISCUSSION** |  | **DISCUSSION** |  |
| Summary of evidence | 19 | Summarize the main results, including an overview of concepts, themes, and types of evidence, and relate them to the review questions and objectives. | Discussion: Principal Findings; Comparison With Previous Research; Clinical and Methodological Implications; Large Language Models and Multimodal AI; Equity and Implementation Considerations. |
| Limitations | 20 | Discuss the limitations of the scoping review process. | Discussion: Limitations. |
| Conclusions | 21 | Provide a general interpretation of the results with respect to the review questions and objectives, as well as potential implications and next steps. | Discussion: Recommendations for Future Research; Conclusions; Abstract: Conclusions. |
| **FUNDING** |  | **FUNDING** |  |
| Funding | 22 | Describe sources of funding for the included sources of evidence and sources of funding for the scoping review, and describe the role of funders. | Funding statement. The manuscript states that the review received no specific grant from any funding agency in the public, commercial, or not-for-profit sectors. |
